# A novel hierarchical biofunctionalized 3D-printed porous Ti6Al4V scaffold with enhanced osteoporotic osseointegration through osteoimmunomodulation

**DOI:** 10.1186/s12951-022-01277-0

**Published:** 2022-02-05

**Authors:** Wei Wang, Yinze Xiong, Renliang Zhao, Xiang Li, Weitao Jia

**Affiliations:** 1 Department of Orthopedic Surgery, Shanghai Jiao Tong University Affiliated Sixth People’s Hospital, Shanghai, 200233 China; 2 Department of Orthopedic Surgery, and Shanghai Institute of Microsurgery on Extremities, Shanghai Jiao Tong University Affiliated Sixth People’s Hospital, Shanghai, 200233 China; 3 School of Mechanical Engineering, State Key Laboratory of Mechanical System and Vibration, Shanghai Jiao Tong University, Shanghai, 200240 China

**Keywords:** 3D-printed Ti6Al4V, MOF, Hierarchical biofunctionalization, Macrophage polarization, Osteogenic–osteoclastic differentiation, Osteoporotic osseointegration

## Abstract

**Background:**

Femoral stem of titanium alloy has been widely used for hip arthroplasty with considerable efficacy; however, the application of this implant in patients with osteoporosis is limited due to excessive bone resorption. Macrophages participate in the regulation of inflammatory response and have been a topic of increasing research interest in implant field. However, few study has explored the link between macrophage polarization and osteogenic–osteoclastic differentiation. The present study aims to develop a novel hierarchical biofunctionalized 3D-printed porous Ti6Al4V scaffold with enhanced osteoporotic osseointegration through immunotherapy.

**Method:**

To improve the osteointegration under osteoporosis, we developed a hierarchical biofunctionalized 3D-printed porous Ti6Al4V scaffold (PT). Biomimetic extracellular matrix (ECM) was constructed inside the interconnected pores of PT in micro-scale. And in nano-scale, a drug cargo icariin@Mg-MOF-74 (ICA@MOF) was wrapped in ECM-like structure that can control release of icariin and Mg^2+^.

**Results:**

In this novel hierarchical biofunctionalized 3D-printed porous Ti6Al4V scaffold, the macroporous structure provides mechanical support, the microporous structure facilitates cell adhesion and enhances biocompatibility, and the nanostructure plays a biological effect. We also demonstrate the formation of abundant new bone at peripheral and internal sites after intramedullary implantation of the biofunctionalized PT into the distal femur in osteoporotic rats. We further find that the controlled-release of icariin and Mg^2+^ from the biofunctionalized PT can significantly improve the polarization of M0 macrophages to M2-type by inhibiting notch1 signaling pathway and induce the secretion of anti-inflammatory cytokines; thus, it significantly ameliorates bone metabolism, which contributes to improving the osseointegration between the PT and osteoporotic bone.

**Conclusion:**

The therapeutic potential of hierarchical PT implants containing controlled release system are effective in geriatric orthopaedic osseointegration.

**Graphical Abstract:**

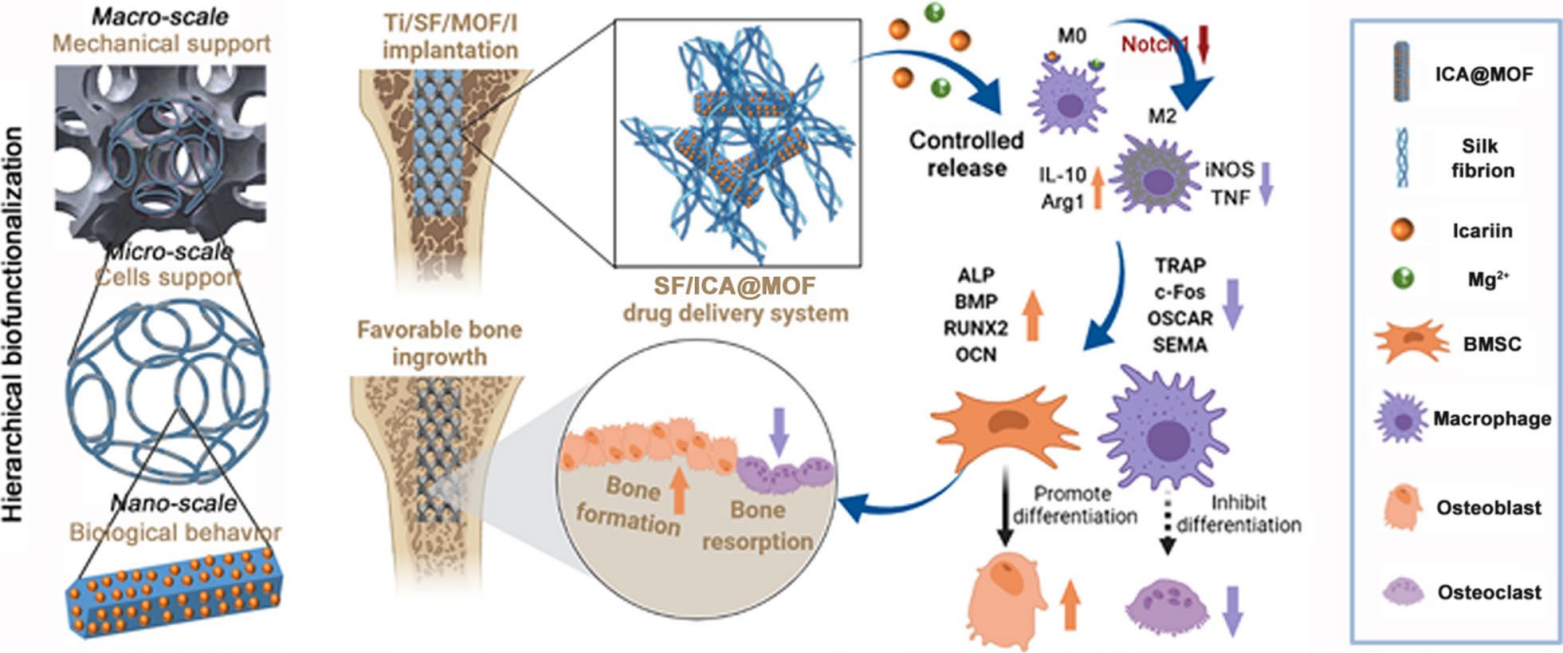

**Supplementary Information:**

The online version contains supplementary material available at 10.1186/s12951-022-01277-0.

## Introduction

With the ageing population, osteoporotic prosthesis loosening has become a major clinical challenge [1]. Due to the decrease in bone density and quality caused by abnormal bone metabolism, brittle fracture and bone defects will occur in patients suffering from low-energy injury, especially hip fracture in elderly patients, which seriously threatens their life and health [1, 2]. Because osteoclast-mediated bone resorption overwhelms the bone regeneration process induced by osteoblasts, complications such as prosthesis loosening and fracture caused by poor osteointegration are more likely to occur in patients with hip arthroplasty, leading to implant failure [3, 4]. This causes great pain and economic burden to patients and families, so the treatment of osteoporotic integration is a difficult problem in urgent need of a solution.

Porous titanium (PT), especially 3D-printed PT, is widely recognized as possessing favourable osteointegration and can stimulate bone ingrowth because of its rationally designed porous structure and low elastic modulus [5–7]. Thus, PT is a promising material for osteoporotic defect repair. However, preliminary clinical trials of PT implants showed that although bone ingrowth was observed inside the PT, the depth of bone ingrowth was limited and occurred only near the implant/bone interface [8]. Predictably, the osteointegration advantage of PT will be reduced under osteoporotic conditions, weakening the clinical benefits of PT implants. Therefore, regulating bone metabolism is a key need in next-generation osteoporotic implants [9]. An ideal osteoporotic implant could not only act as a mechanical supporting material, but also actively participate in the bone regeneration process under osteoporotic conditions [10]. As a bioinert material, PT requires biofunctionalized modification to perform this function. Pharmacological agents are typical clinical treatments for osteoporosis, and icariin, an extract used in traditional Chinese medicine, has exhibited promising in vivo outcomes in treating osteoporosis [11–13]. Moreover, PT has an interconnected pore structure with a large surface area, which provides favourable prerequisites for constructing local drug delivery systems in/on it [14]. The local drug delivery system can directly influence the microenvironment in the implantation area and effectively regulate bone metabolism. Therefore, it has recently attracted considerable interest.

Previously published studies on developing local drug delivery systems on implants used for osteoporotic applications were based on surface modification [15]. However, due to the complex pore structure and required pore size (~ 500 μm) of PT, conventional surface modification methods cannot form uniform coatings on the surface of PT, especially in its core area. Moreover, bone implants are usually irregular in shape, which further increases the difficulty of modification processes. Davoodi et al. developed a method for the biofunctionalization of PT by embedding cell-laden gelatin methacryloyl hydrogels, and in vitro results showed improved biological responses [16]. Yavari et al. proposed a method for the biofunctionalization of PT that involves layer-by-layer coating with gelatin incorporating BMP2 and vancomycin [17]. These previous studies provide new insight into developing local drug delivery systems for PT. However, using the biodegradability of hydrogels to release anti-osteoporosis drugs can lead to burst release problems and consequently result in limited clinical outcomes. However, osseointegration is a long-term process that requires precise and long-term drug release in the implant to achieve long-term effects [18]. Therefore, it remains a major challenge to develop a controlled-release drug delivery system in PT.

Recent advances in nanomaterials provide opportunities for developing controlled-release drug delivery systems [19–21]. Among them, metal–organic frameworks (MOFs) have attracted much interest due to their designable composition, chemical properties, large surface areas, biodegradability, and high porosity, which make them promising platforms for drug delivery [22, 23]. Hu et al. investigated the drug delivery mechanisms of MOF-74 and showed that MOF-74 had low cytotoxicity and controllable drug release; thus, it is a promising drug host [24]. Shen et al. constructed a Mg/Zn-MOF coat on a titanium surface and suggested that the Zn^2+^ and Mg^2+^ released during the biodegradation of MOF can improve new bone formation and exert antibacterial effects [25]. Therefore, Mg-MOF-74 is a promising drug delivery cargo for orthopaedic applications that has seldom been studied before. However, MOFs are not stable in acidic environments, and the microenvironment surrounding an implant is usually acidic, so further modification of MOFs is needed for orthopaedic application.

In our previous study, we developed a PT/silk fibroin (SF) composite scaffold. SF can form an extracellular matrix (ECM)-like structure inside PT after freeze-drying and thereby provide a favourable microenvironment for cell adhesion and proliferation [26]. In addition, due to the excellent biodegradability of SF, this composite scaffold can be used as a drug delivery platform [27, 28]. Therefore, based on the proposed SF-constructed ECM-like structure, the drug-loaded MOF can be incorporated into PT and protected by SF. Presumably, this synergistic drug delivery system could endow PT with the ability to control the release of anti-osteoporosis drugs, which has not been previously reported.

In addition, previous studies have focused on the direct osteogenic differentiation of stem cells with PT implants. Currently, increasing attention has been given to the inflammatory reaction between tissues and implanted biomaterials [29, 30]. Macrophages, including progenitor cells and monocytes, are the first line of defence against foreign implants [31]. Increasing evidence shows that the type of macrophage response plays an important role in mediating the tissue repair of scaffolds [31]. Some studies have shown that cytokines secreted by macrophages, such as IL-4, IL-10, and IL-13, can promote bone formation, while proinflammatory factors, such as TNF, IL-1, and IL-6, enhance bone resorption [32]. In addition, macrophages are the main precursors of osteoclasts [33]. Therefore, in addition to directly regulating osteoblast differentiation and inhibiting osteoclast differentiation at PT implants, it is possible to optimize the osteointegration of PT implants under the condition of osteoporosis by simultaneously regulating the immune response of macrophages.

In this study, a novel hierarchical biofunctionalized 3D-printed porous Ti6Al4V scaffold with macro/micro/nano-scales was developed and systematically investigated. The mechanisms of the proposed biofunctional method and regulation of the macrophage response for osteoporotic osseointegration were revealed by in vitro and in vivo experiments. This work will generate fresh insight into the development of orthopaedic implants for osteoporotic osseointegration.

## Materials and methods

### Preparation of 3D-printed PT

**Fig. 1:**
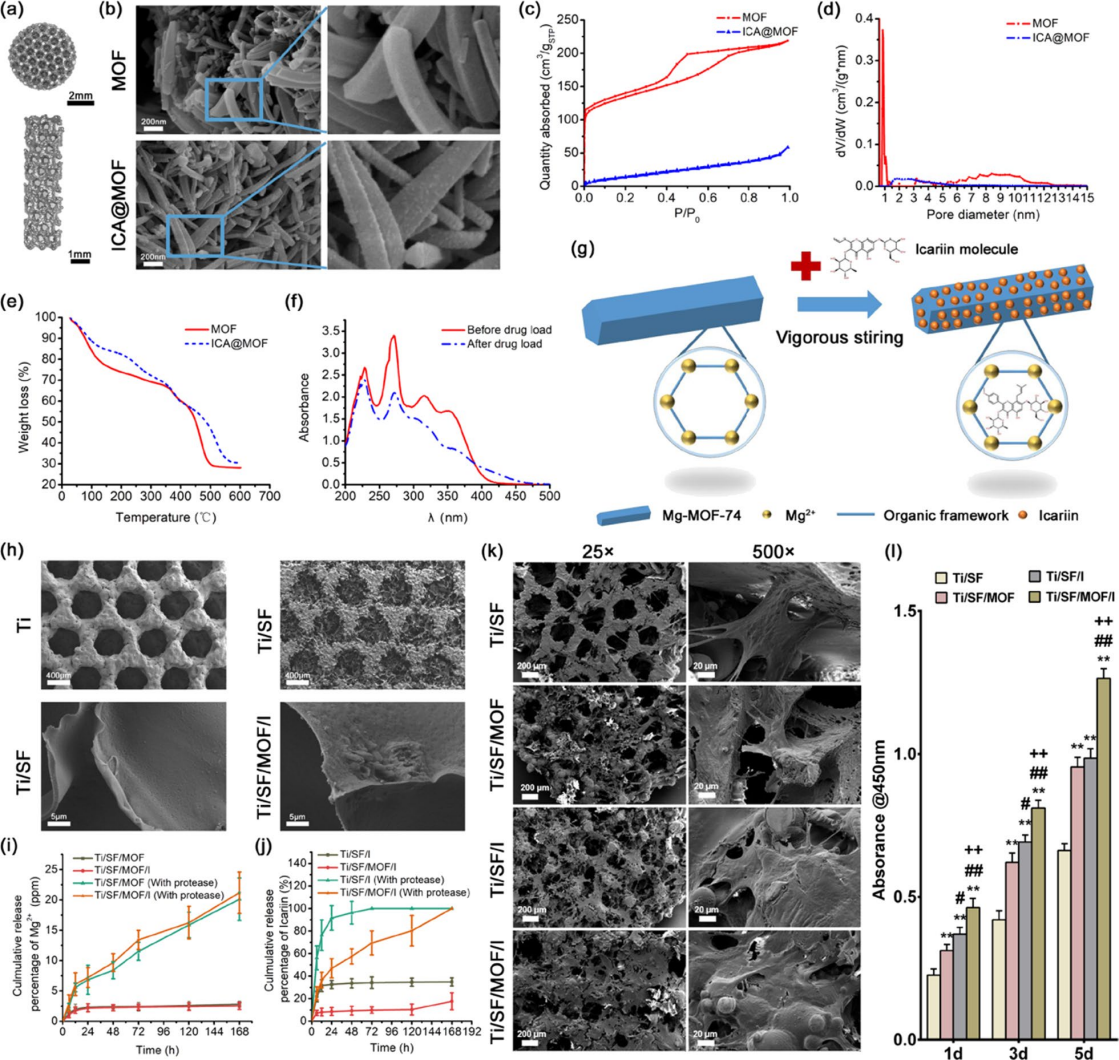
Characterization and biocompatibility of different samples. **a** Different sizes of 3D-printed PT scaffolds. **b** The morphology of MOF before and after icariin-loading. **c** N_2_ physisorption isotherms of MOF before and after icariin-loading. **d** The pore size distribution of MOF before and after icariin-loading. **e** The thermogravimetric analysis results of the MOF before and after icariin-loading. **f** UV–vis absorption spectra of the icariin solution before and after MOF loading. **g** Schematic diagram of the icariin loading process of MOF. **h** The microscopic morphology of the different samples by SEM. **i**, **j** Icariin release curve and Mg^2+^ release curve of each group of biofunctionalized PT scaffolds in protease-free and protease-included PBS for different time periods. **k** Adhesion morphology of rBMSCs on each group of scaffolds. **l** The CCK-8 assay assesses the proliferation of Raw264.7 cells on porous scaffolds. (n = 3; *, # and + represent P < 0.05 when compared with Ti/SF, Ti/SF/MOF and Ti/SF/I, respectively; **, ## and ++ represent P < 0.01)

The model of PT was constructed by triply periodic minimal surface (TPMS), the unit cell was a TPMS-diamond structure, and the designed pore size and porosity were 500 μm and 75%, respectively. Three PT objects were designed in this study, all of which were porous cylinders. A 5 mm × 2 mm (diameter × height) PT was designed for in vitro testing, a 2 mm × 8 mm PT was designed for the in vivo experiments (Fig. 1a), and a 10 mm × 10 mm PT was built for mechanical testing. Equation (1) was used for constructing the TMPS.

1 $$\cos \left( {1.6\pi x} \right) \cdot \cos \left( {1.6\pi y} \right) \cdot \cos \left( {1.6\pi z} \right) - {\text{sin}}\left( {1.6\pi x} \right) \cdot {\text{sin}}\left( {1.6\pi y} \right) \cdot {\text{sin}}\left( {1.6\pi z} \right) > 0.4243$$

Using Eq. (1), the STL file for 3D printing was built in Mathematica (Wolfram Research, USA). Then, the designed STL model was imported for manufacturing. Additive manufacturing processes were conducted by selective laser melting (SLM) technology with an EOS M290 SLM machine (Munchen, Germany). The raw material was Ti6Al4V powders (20–50 μm, EOS GmbH, Germany). The SLM fabrication parameters were set as follows: laser power of 280 W, laser spot diameter of approximately 80 μm, scan speed of 1200 mm/s and layer thickness of 30 μm. SLM printing was conducted in an argon atmosphere to prevent oxidation. After additive manufacturing, all SLM-printed PTs were cleaned with compressed air and an ultrasonic cleaner.

### Preparation of ICA@MOF and biofunctionalized PT

Mg-MOF-74 was dried under vacuum conditions at 100 ℃ for 1 h. The dried MOF (300 mg) was then added to icariin (Solarbio, China) [10 mg/mL in 10 mL DMSO (Sinopharm Chemical Reagent, China)] solution and vigorously stirred at 60 ℃ for 2 days. The resultant crystals were filtered, washed with methanol, ethanol and deionized water, and dried overnight under vacuum conditions to obtain an icariin-containing MOF solid (ICA@MOF). The ICA/DMSO solution before adding MOF and the filtrate after drug loading were retained for drug loading analysis.

The hierarchical biofunctionalization of PT was performed based on our previous study [26]. Briefly, SLM-printed PTs were placed in a 72-well plate. Four types of solutions were prepared: (a) 5 wt.% SF (Simatech, China) aqueous solution, (b) 1 mg/mL icariin in 5 wt.% SF aqueous solution, (c) 4 mg/mL Mg-MOF-74 in 5 wt.% SF aqueous solution, and (d) 4 mg/mL ICA@MOF in 5 wt.% SF aqueous solution. The SF solution supplemented with icariin, MOF or ICA@MOF was stirred until a homogeneous suspension was formed. Afterwards, the four types of solutions were slowly injected into the PT in 72-well plates until the PTs were completely submerged by the solution. The plate was placed in a refrigerator, frozen at – 25 ℃ for 12 h and then lyophilized for 48 h at 0.02 Torr and – 80 ℃ by a freeze dryer (Freezone 6PLUS, Labconco, USA). After complete drying, 75% ethanol/water solution was added to each well to denature the SF to a water-insoluble material. The freezing-lyophilization process was repeated, and four groups of samples were achieved: (a) PT/SF (Ti/SF), (b) PT/SF/MOF (Ti/SF/MOF), (c) PT/SF/icariin (Ti/SF/I), and (d) PT/SF/ICA@MOF (Ti/SF/MOF/I). The group number corresponds to the type of solution added to the PT.

### Sample characterization

#### Mechanical properties of ICA@MOF-PT

The mechanical properties of PT and biofunctionalized PT were measured by quasi-static compression tests according to the standard ISO13314 for compression tests of porous and cellular metals. A universal testing machine (MTS 809, USA) was used in this study. The loading rate was set to 0.5 mm/min, and three duplicate samples were tested for each group. During the compression process, the compressive force and displacement were documented, and the stress–strain curves of the samples were plotted by OriginPRO (OriginLab, USA). The elastic modulus E and yield strength σ_y_ of the samples were calculated according to the stress–strain curves.

#### Characterization of ICA@MOF

Scanning electron microscopy (SEM, Zeiss Ultra Plus, Carl Zeiss co, Germany) was employed to analyse the morphology of the MOF and ICA@MOF. The amount of icariin adsorbed into the porous microcrystal was measured by UV–Vis spectroscopy (EV300, Thermo Fisher, USA) and was estimated at 270 nm according to the standard curve of icariin. The solutions before and after drug loading were diluted by a factor of 50 before the test. The microstructure of ICA@MOF was further investigated by thermogravimetric analysis (TGA, STA 449 F3, Netzsch, Germany) and specific surface area and porosity analysis (Autosorb-IQ3, MicrotracBEL, Japan). The heating rate was 5 ℃/min, and heating was carried out between 30 and 700 ℃ under an air atmosphere for all TGA tests. The surface area and porosity of the porous solid were determined by N_2_ adsorption using the BJH method.

#### Characterization of biofunctionalized samples

Sample dimensions of 5 mm × 2 mm (diameter × height) were adopted in the following investigation. Microscopic images of PT, Ti/SF, Ti/SF/MOF, Ti/SF/I, and Ti/SF/MOF/I were visualized by SEM (Zeiss Ultra Plus, Carl Zeiss co, Germany).

To assess the drug release behaviour of icariin from Ti/SF/I and Ti/SF/MOF/I, the samples were submerged in PBS solutions (5 mL, pH = 5.5) and incubated on a shaker at 50 rpm and 37 ℃ for different durations (0.25, 0.5, 1, 2, 3, 5 and 7 days). Three duplicate samples were prepared for each group, and protease XIV (Sigma-Aldrich, USA) (0.5 mg/mL) was added to the solution in three of them. The solution was then removed, and a UV–Vis spectrophotometer (EV300, Thermo Fisher, USA) was used to assess the amount of icariin released, which was calculated according to the standard curve of icariin.

To measure the concentration of Mg^2+^ ions released from Ti/SF/MOF and Ti/SF/MOF/I, the specimens were immersed in PBS (5 mL, pH = 5.5) and shaken at 50 rpm and 37 ℃ for different durations. Six duplicate samples were prepared for each group, and protease XIV (0.5 mg/mL) was added to the solution for three of them. The supernatant solution was then collected to measure the Mg^2+^ ion concentration by an inductively coupled plasma-atomic emission spectrometer (ICP-AES, iCAP7600, Thermo Fisher, USA).

### Cell experiments

#### Cell culture

Mouse macrophage cells (Raw264.7) were purchased from the Chinese Academy of Science cell bank. Mouse bone marrow macrophages (BMMs) and rat bone marrow mesenchymal stem cells (rBMSCs) were extracted and cultured in MEM-α (HyClone) containing 10% foetal bovine serum (FBS, Gibco) and 1% penicillin/streptomycin (Gibco) according to previous study [34, 35]. After reaching 80% fusion, Raw264.7/BMMs were passed through a cell scraper, while rBMSCs were subjected to trypsin digestion.

#### Cell adhesion and proliferation on samples

rBMSCs (5 × 10^4^) were seeded on scaffolds for 1 day. The cells/scaffolds were then dehydrated with ethanol and dried in a freeze dryer. Finally, the cells/scaffolds were sputter-coated with gold and observed by SEM. The CCK-8 assay was employed to assess the proliferation of Raw264.7 on scaffolds. The cells were inoculated on scaffolds at a rate of 5 × 10^4^ per well in 96-well plate. After culturing for 1, 3 and 5 days, the cells/scaffolds were rinsed with PBS and cultured in medium containing 10% CCK-8 for 4 h at 37 °C, and then a spectrophotometer (Bio-Rad, USA) was used to obtain the absorbance at 450 nm. Live/dead assay was also used to examine the cytotoxicity toward extracts of the different kinds of samples. Raw264.7 were seeded at a density of 2 × 10^5^ cells/well in 24-well plate. After incubation for 1 and 5 days, the Raw264.7 cells were stained with calcein-AM (Sigma, USA) and PI (Sigma, USA) solution, and images of the live/dead test were collected under an immunofluorescence microscope (Leica). For the preparation of the extract, a disk sample (5 mm × 2 mm) was immersed in 1 mL of MEM-α with 10% FBS and 1% penicillin/streptomycin in a cell incubator for 72 h. The extracts were filtered with a 0.22 μm filter and placed in a 4 °C refrigerator.

#### In vitro polarization of Raw264.7 cells cultured in sample extracts

After continuous culturing in sample extracts for 4 days, the cell medium was collected and centrifuged. The concentrations of TNF-α, IL-4, IL-6, and IL-10 in the supernatants were determined by ELISA kits (Elabscience Biotechnology Co., Ltd.) according to the manufacturer’s instructions.

Fluorescence staining was employed to assess the expression levels of the M1 marker iNOS (green) and M2 marker Arg-1 (red). After incubation with extract for 4 days, Raw264.7 cells were fixed, permeabilized, blocked, and incubated with primary antibodies against iNOS (1:100, Abcam) and Arg-1 (1:50, Abcam) overnight at 4 °C. The secondary antibodies donkey anti-rabbit Alexa Fluor 488 (1:200, Abcam) and donkey anti-mouse Alexa Fluor 594 (1:200, Abcam) were allowed to bind the primary antibody for 2 h. Finally, the nuclei were stained blue with DAPI (Solarbio, China) and observed with a fluorescence microscope (Leica).

The expression of M1 (chemokine receptor CCR7) and M2 (mannose receptor CD206) macrophage surface markers was further determined by flow cytometry. After incubation with extract for 4 days, Raw264.7 cells were blocked by 1% BSA and incubated with primary antibodies against CCR7 (1:100, BD Pharmingen, Alexa Fluor 647) and CD206 (1:100, BioLegend, Alexa Fluor 488) at 4 °C for 30 min. The cells were analyzed on flow cytometer (BD FACSAria). The data were analyzed using FlowJo version 10.4 software (FlowJo, LLC, Ashland, Oregon, USA).

RT-qPCR was also carried out to examine the expression levels of M1 macrophage marker (iNOS and TNF-α) and M2 macrophage marker (Arg-1 and IL-10). Raw264.7 cells were cultured in extract for 4 days to extract RNA and obtain complementary DNA (cDNA) as described in our previous study [35], and RT-qPCR analysis was performed using SYBR Green detection reagents. GAPDH was used as an internal control for normalization. All experiments were repeated three independent times. The formula 2^−△△Ct^ was applied to calculate the relative expression of mRNAs. The primer information is provided in Additional file 1: Table S1.

#### ICA@MOF regulates macrophage polarization by inhibiting the Notch1 pathway

Fluorescence staining was employed to assess the expression level of Notch1 in Raw264.7 cells. After incubation with extract for 4 days, Raw264.7 cells were fixed, permeabilized, blocked, and incubated with primary antibodies against Notch1 (1:100, Abcam) overnight at 4 °C. The secondary antibody donkey anti-mouse Alexa Fluor 594 (1:200, Abcam) were allowed to bind the primary antibody for 2 h. Finally, the nuclei were stained blue with DAPI (Solarbio, China) and observed with a fluorescence microscope (Leica).

Western blotting was further applied to evaluate the protein expression of Notch1. The cells were washed three times with PBS, and the total protein was extracted, measured, separated, transferred and blocked according to the manufacturer’s instructions. All PVDF membranes were cut into several small strips using the protein markers as a reference, and primary antibody against Notch1 (1:1000, Abcam), and β-actin (1:10,000, ABclonal) were added and incubated with the corresponding membrane strips at 4 °C overnight. The following day, the membranes were washed several times with Tris-buffered saline plus Tween-20 (TBS-T) and then incubated with secondary antibodies (1:1000) for 1 h. Finally, enhanced chemiluminescence reagent (Beyotime) was used to image the target protein signals, and a ChemiDoc CRS imaging system (Bio-Rad, USA) was used to detect the proteins.

#### Osteogenic differentiation evaluation of Raw264.7-conditioned medium

Conditional medium was obtained by culturing Raw264.7 cells on different scaffolds for 4 days, collecting the supernatants and mixing them with MEM-α at a ratio of 1:2 [36]. rBMSCs were cultured with MEM-α at a density of 1 × 10^4^ cells per well in a 24-well plate. After incubation for 12 h, the medium was replaced with conditioned medium for further culture. On days 7 and 14, the cells were tested with ALP staining/activity kits (Beyotime) or Alizarin red (Cyagen). Quantitative analysis of ALP activity and Alizarin red staining was conducted according to our previous studies [37]. Further, we determined the early and later osteogenic differentiation protein, ALP and osteocalcin (OCN), by immunofluorescence staining at day 7 and 14, respectively. The nuclei was stained with DAPI (Sigma-Aldrich, USA). An ALP antibody (1:200, Abcam108337) and OCN antibody (1:200, Abcam13420) were stained green by the corresponding antibody, respectively [37]. The expression of osteogenic genes in rBMSCs detected by RT-qPCR was performed as described in “In vitro polarization of Raw264.7 cells cultured in sample extracts” section. The primer information is provided in Additional file 1: Table S2. The expression of osteogenic proteins in rBMSCs detected by western blotting was performed as described in “ICA@MOF regulates macrophage polarization by inhibiting the Notch1 pathway” section. Proteins of ALP (Affinity DF6225), BMP-2 (Affinity AF5163), RUNX2 (Affinity AF5186) and OCN (Affinity DF12303) were detected, and GAPDH (Affinity AF7021) was used as internal control.

#### Osteoclastic differentiation evaluation of Raw264.7-conditioned medium

The supernatants of culture medium and MEM-α complete medium were prepared at a 1:2 ratio with 30 ng/mL M-CSF (Pepro Tech, USA) and 100 ng/mL RANKL (Pepro Tech, USA). BMMs were inoculated into 24-well plates at a density of 5 × 10^5^ cells/well and cultured in MEM-α complete medium containing 30 ng/mL M-CSF for 1 day and the medium was replaced by conditioned medium for further incubation. When large multinucleated cells had formed, the cells were fixed in 4% paraformaldehyde for 15 min, and then tartaric acid-resistant staining kits (Sigma-Aldrich, USA) were used for TRAP staining. To evaluate whether the formation of bone resorption and podosome belts in mature osteoclasts were affected by the conditioned medium, Corning Osteo assay and fluorescence staining of F-actin rings were also performed as previously described. The expression of osteoclastic genes detected by RT-qPCR was performed as described in “In vitro polarization of Raw264.7 cells cultured in sample extracts” section. The primer information is provided in Additional file 1: Table S3.

### In vivo experiments

#### Animals and models

Based on previous study [36], an air pouch was first developed on the backs of C57BL/6 mice by injecting sterile air subcutaneously. The mice were then anaesthetized via intraperitoneal injection of 0.5% pentobarbital (9 mL/kg), and the skin of the air pouch was shaved an disinfected. An incision was made proximal to the pouch, one scaffold (5 mm × 2 mm) was inserted into the pouch, and the incision was sutured carefully. In total, 12 C57BL/6 mice were used for experiments (n = 3 for each group).

The animal model of osteoporosis was established by bilateral ovariectomy in 12-week-old female SD rats. The rats were anaesthetized by intraperitoneal injection of 0.5% pentobarbital (9 mL/kg), and the back was shaved and disinfected. An incision was made on both sides of the spine and the ovaries were exposed and excised and sutured. The osteoporotic model was established 12 weeks after surgery. The rats were anaesthetized by intraperitoneal injection of 0.5% pentobarbital (9 mL/kg), and the knee joint was shaved and disinfected. A 2 mm diameter Steinmann pin was opened parallel to the long axis of the femur, and a 2 mm × 8 mm cylindrical sample was inserted for 8 weeks. At 2, 4 and 6 weeks postoperatively, the new bone was labelled by intraperitoneal injection of tetracycline, calcein and alizarin red [35]. In total, 12 female SD rats were used for experiments (n = 3 for each group).

#### In vivo immunomodulatory evaluation

Four days after the sample was implanted into the air pouch, the mouse was sacrificed. The skin covering the implants was harvested and fixed with paraformaldehyde. Further tissue sections were made after embedding in paraffin. Then, the sections were stained with haematoxylin and eosin (H&E) to assess the inflammatory reaction of the skin. The intensity of macrophages with different phenotypes in the fibrous layer was quantified by immunofluorescence staining with the same antibody used in the in vitro immunofluorescence staining for macrophages.

#### Micro-CT analysis

Eight weeks after surgery, the rats were sacrificed by an overdose of pentobarbitone sodium. The femurs containing the implants were harvested, subjected to micro-CT scanning, and analysed as described in a previous study [36].

#### Evaluation of osteoporotic bone integration by undecalcified sections

After micro-CT analysis, the femurs were embedded in polymethylmethacrylate (PMMA), and undecalcified sections were acquired using a Leica SP1600. A confocal laser scanning microscope (Leica CLSM) was applied to detect tricolour-labelled bone tissue. Finally, the sections were stained with acid fuschin and observed by an optical microscope to reveal the integration between porous implant and host bone under the pathological condition of osteoporosis.

### Statistical analysis

All data are presented as the mean ± SD. Differences among groups were analysed with one-way ANOVA followed by the Student–Newman–Keuls test using SPSS 17.0 software, and P < 0.05 was considered statistically significant.

## Results

### Preparation of drug delivery cargo

As shown in Fig. 1b, the MOF was a prismatic nanostructure with a smooth surface. In contrast, the surface morphology of ICA@MOF was rough and full of bulges, indicating that the drug loading process changed the highly porous crystal structure of the MOF.

According to the nitrogen isotherm assay results in Fig. 1c, d, the adsorption performance and pore size distribution of MOF before and after drug loading were quite different. The nitrogen isotherm for MOF was a typical type for materials with micropores, and the obvious hysteresis loop implied that the micropores consist of relatively large holes and narrow slit-like holes, which was consistent with the framework of MOF-74 depicted by Deng et al. [38]. After drug loading, the adsorption ability of ICA@MOF decreased significantly, and the hysteresis loop disappeared. From the pore size distribution results, the pore size of 1 nm was gone, and the pore size of 6–11 nm was shifted to 1.5–5 nm after the drug loading. The results indicated that the pores of the MOF, especially the narrow slit-like holes, were filled or blocked by some molecules after drug loading.

To define the molecules in the pores of MOF, TGA analyses of MOF and ICA@MOF were carried out. The weight loss curves in Fig. 1e showed two obvious weight losses in both MOF and ICA@MOF. The first weight loss (25–100 °C) was because of the departure of water and the second weight loss (350–500 °C) was due to the destruction of the MOF. Because the boiling point of icariin was approximately 948.5 °C and no other materials were included during the drug loading process, it can be concluded that the incorporated icariin was the cause of the weight loss difference between MOF and ICA@MOF. Thus, the results indicated that the molecules blocking the pore structure of MOF are icariin.

To estimate the loading percentage of the MOF, UV–vis adsorption spectra plots of the icariin solution before and after drug loading were shown in Fig. 1f. The absorbance at 270 nm was decreased after drug loading, indicating that icariin was absorbed into the pore structure of the MOF. The drug content absorbed into the MOF was obtained from the standard curve, and the drug loading percentage was calculated as 12.4%. The loading percentage was comparable to previously published results using MOF as a drug delivery cargo [22]. Figure 1g showed the schematic diagram of icariin adsorption by MOF structure to form ICA@MOF.

### Characterization of biofunctionalized PT

The mechanical properties of PT before and after biofunctionalization were obtained from the stress–strain curves shown in Additional file 1: Fig. S1. The elastic modulus and yield strength of PT and Ti/SF/MOF/I were 3.43 ± 0.05 GPa and 71.16 ± 3.18 MPa, 3.39 ± 0.04 GPa and 71.42 ± 2.47 MPa, respectively. The results indicated that the biofunctionalization processes did not affect the mechanical behaviour of PT.

The SEM images of the prepared samples were shown in Fig. 1h. The pore size of PT was approximately 500 μm and its porosity was approximately 73.6%. As shown in Fig. 1h, the SF solution formed a highly porous uniform network structure inside the pore structure of PT after lyophilization and 75% ethanol denaturation. The pore size of the SF network was larger than 100 μm.

For Ti/SF/MOF/I, ICA@MOF wrapped by SF can be observed on the SF network structure inside the PT (Fig. 1h). Therefore, the SF network can also act as a platform for biofunctionalization, and effectively realize the load of ICA@MOF in PT.

The drug release tests further demonstrated the validity of the as-built controlled-release system (Fig. 1i, j). The MOF and icariin were stable in the SF network, and only small amounts of icariin and Mg^2+^ were released from the scaffold. However, with the degradation effect of protease, the MOF wrapped in SF will gradually release Mg^2+^ from the scaffold, as shown in Fig. 1i. Regarding the release behaviour of icariin (Fig. 1j), burst release was observed in the Ti/SF/I group. However, Ti/SF/MOF/I exhibited a controlled release of icariin, indicating that wrapping ICA@MOF with SF can reduce the rate of MOF disassembly and hence achieve the controlled release of icariin in PBS (pH = 5.5). Bone tissue regeneration requires a long period of time. Therefore, the controlled release of icariin and Mg^2+^ can act more effectively on the microenvironment of bone regeneration.

### In vitro biocompatibility of Ti/SF/MOF/I

To determine the biocompatibility of samples, both rBMSCs and Raw264.7 were seeded on samples in vitro. The SEM images in Fig. 1k displayed that rBMSCs adhered well on the modified porous surfaces of all four groups of scaffolds. The CCK-8 assay was performed to assess the proliferation of Raw264.7 cells on porous scaffolds, and the results displayed that Ti/SF/MOF/I afforded the highest cell viability (Fig. 1l). In addition, live and dead staining results also showed that the scaffolds extracts had no toxicity to Raw264.7 cells, which was consistent with CCK-8 test (Additional file 1: Fig. S2). According to the SEM images and CCK-8 analyses, we concluded that among the scaffolds tested, Ti/SF/MOF/I possessed the best biocompatibility.

### In vitro evaluation of macrophage polarization

**Fig. 2:**
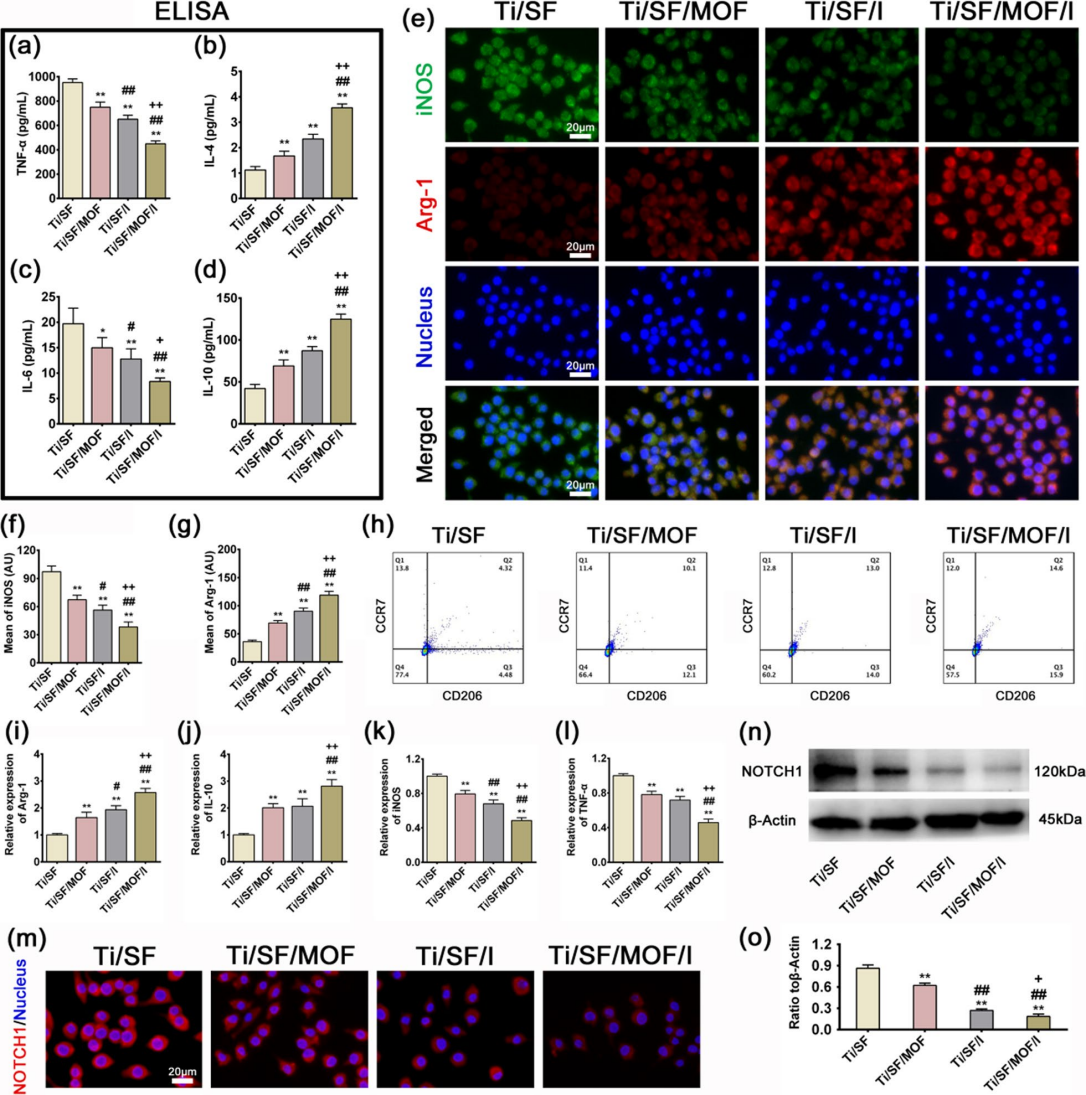
In vitro evaluation of Raw264.7 cells polarization. **a**–**d** ELISA results of TNF-ɑ, IL-4, IL-6, and IL-10 respectively. **e** Immunofluorescent staining of Raw264.7 cells after cultured for 4 days. **f**, **g** Quantitative analysis of iNOS and Arg-1. **h** The polarization of Raw264.7 cells was analyzed by flow cytometry. **i**–**l** RT-qPCR results of Arg-1, IL-10, iNOS, and TNF-ɑ after Raw264.7 cells cultured for 4 days. **m** Immunofluorescent staining of Notch1. **n** The expression of Notch1 was detected by western blotting. **o** The expression of Notch1 protein was quantitatively analyzed by Image J. (n = 3; *, # and + represent P < 0.05 when compared with Ti/SF, Ti/SF/MOF and Ti/SF/I, respectively; **, ## and ++ represent P < 0.01)

To evaluate the representative cytokines secreted by M1 and M2 macrophages, ELISA was applied to detect the concentrations of TNF-α, IL-4, IL-6, and IL-10. The results were presented in Fig. 2a–d. Raw264.7 in Ti/SF/MOF/I secreted the highest amounts of the anti-inflammatory cytokines IL-4 and IL-10, which are mainly produced by M2 macrophages. In contrast, the expression levels of two inflammatory cytokines, TNF-α and IL-6, were the highest in Ti/SF.

To further validate the polarization of macrophages, immunofluorescence staining was used to monitor iNOS (green, M1 marker) and Arg-1 (red, M2 marker) in Raw264.7 cultured for 4 days. Clearly, the Ti/SF/MOF/I group featured a higher intensity of Arg-1-positive cells than the other groups (Fig. 2e, g). iNOS, however, demonstrated the opposite pattern: higher iNOS expression was observed in the Ti/SF group (Fig. 2e, f). To explore how ICA@MOF regulated the Raw264.7 cells polarization, we examined the CCR7 + (M1) and CD206 + (M2) proportion of the Raw264.7 cells by flow cytometry. Compared with the Ti/SF group, all other groups induced the macrophage Raw264.7 cells to transform into more M2 macrophages at day 4, and the Ti/SF/MOF/I, containing ICA@MOF system, induced the fewest M1 (12.05 ± 0.03%) and most M2 (15.92 ± 0.11%) macrophages (Fig. 2h).

To further confirm the ability of Ti/SF/MOF/I to exert immunomodulatory effects, we selected some representative genes to determine their fold changes by RT-qPCR.

First, the typical M2 macrophage markers Arg-1 and IL-10 were upregulated on Ti/SF/MOF/I (Fig. 2i, j), suggesting a higher proportion of M2 macrophages. The RT-qPCR findings were highly consistent with those of the ELISA and the immunofluorescence staining assay.

To reveal the potential mechanism of ICA@MOF regulating macrophage polarization, we detected the expression of Notch1 protein in each group by cellular immunofluorescence and western blotting. As shown in Fig. 2m immunofluorescence staining, the expression of Notch1 was decreased in Ti/SF/MOF and Ti/SF/I groups compared with the Ti/SF group, while the level of Notch1 protein was significantly decreased in the Ti/SF/MOF/I group containing ICA@MOF. The western blotting bands were consistent with cellular immunofluorescence (Fig. 2n, o). These data suggested that ICA@MOF may synergically inhibit the transformation of M0 macrophages into M1 and the release of pro-inflammatory factors by inhibiting Notch1 signaling.

### Osteogenic differentiation effect of macrophage-conditioned medium

To evaluate the immunomodulatory osteogenic effect of samples, macrophage-conditioned medium was prepared. rBMSCs were then cultured in conditioned medium for 7 and 14 days to study their osteogenic differentiation ability.

**Fig. 3:**
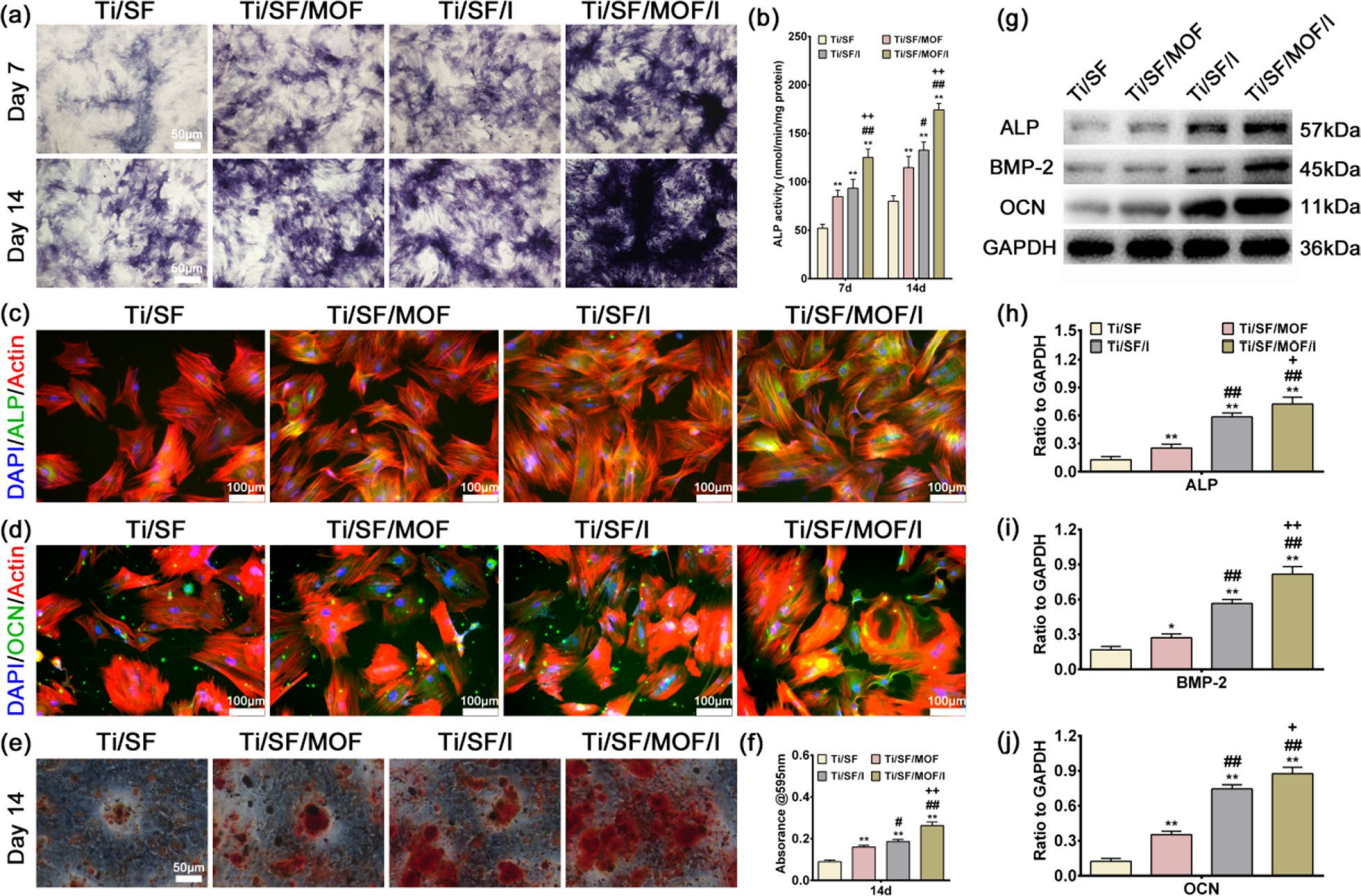
Detection of osteogenic differentiation markers of rBMSCs. **a**, **b** ALP staining and activity of rBMSCs cultured in conditioned medium for 7 and 14 days. **c** ALP immunofluorescent staining of rBMSC in conditioned medium for 7 days: green (ALP), red (actin), blue (Nucleus/DAPI). **d** OCN immunofluorescent staining of rBMSC in conditioned medium for 14 days: green (ALP), red (actin), blue (Nucleus/DAPI). **e** Alizarin Red staining of rBMSC cultured in conditioned medium for 14 days. **f** Quantitative analysis of Alizarin Red staining. **g** ALP, BMP-2 and OCN western blotting bands of rBMSC in conditioned medium for 14 days. **h**–**j** The expressions of ALP, BMP-2 and OCN proteins were quantitatively analyzed by Image J. (n = 3; *, # and + represent P < 0.05 when compared with Ti/SF, Ti/SF/MOF and Ti/SF/I, respectively; **, ## and ++ represent P < 0.01)

Both alkaline phosphate (ALP) staining and immunofluorescence staining were employed to determine ALP expression in the rBMSCs. Figure 3a revealed that over time, ALP expression increased, and the highest ALP expression was observed in the Ti/SF/MOF/I group, followed by the Ti/SF/I and Ti/SF/MOF groups. Consistent with the ALP staining results, similar trends were observed in the ALP activity test and immunofluorescence staining: the highest ALP activity and ALP (green) fluorescence intensity were detected in the Ti/SF/MOF/I group (Fig. 3b, c).

We also detected a later osteogenic differentiation protein, osteocalcin (OCN), by immunofluorescence staining at day 14. The results displayed that cells in the Ti/SF/MOF/I group expressed more OCN (green) than the other groups (Fig. 3d). To study the mineralization level of rBMSCs in the conditioned medium, we conducted Alizarin red staining on day 14. More calcified nodules were stained red in the Ti/SF/MOF/I group than in the other groups (Fig. 3e), which was further confirmed by the quantitative test shown in Fig. 3f. RT-qPCR and western blotting tests were performed to further determine the expressions of osteogenic genes or proteins, i.e., ALP, BMP-2, RUNX2, and OCN, on day 14. The results were shown in Additional file 1: Figs. S3, S4, Fig. 3g–j. rBMSCs in the Ti/SF/MOF/I group expressed the highest level of the selected genes. In summary, the conditioned medium of Ti/SF/MOF/I exerted the strongest effects on osteogenic differentiation, which in turn suggested that the Raw264.7 cells cultured on Ti/SF/MOF/I displayed the best immunomodulatory osteogenic effect.

### Osteoclastic differentiation effect of macrophage-conditioned medium

To evaluate the immunomodulatory osteoclastic effect of samples, BMMs were also cultured with conditioned medium for 5 days to study the ability to inhibit osteoclast differentiation.

**Fig. 4:**
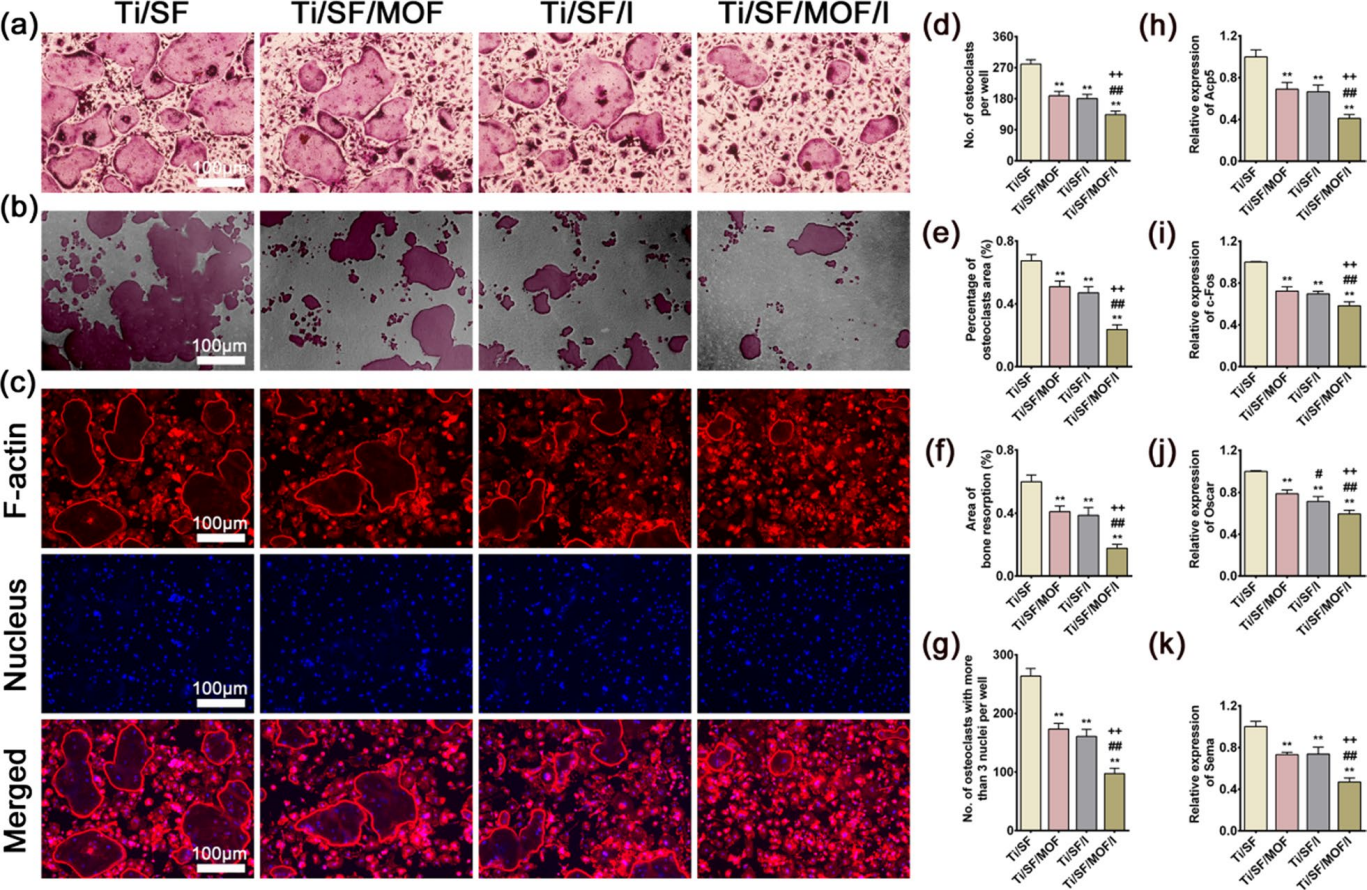
Osteoclastic differentiation effect of Raw264.7 cells conditioned medium. **a** TRAP staining of BMMs cultured in conditioned medium for 5 days. **b** The bone resorption assay of BMMs cultured in conditioned medium for 5 days: claret indicates eroded area. **c** F-actin rings staining of BMMs cultured in conditioned medium for 5 days. **d**, **e** Quantitative analysis of the number and area of osteoclasts in TRAP staining. **f** Quantitative analysis of the eroded area in bone resorption assay. **g** Quantitative analysis the total number of osteoclasts with more than three nuclei in F-actin rings staining. **h**–**k** RT-qPCR results of Acp5, c-Fos, Oscar, and Sema after BMMs cells cultured in conditioned medium for 5 days. (n = 3; *, # and + represent P < 0.05 when compared with Ti/SF, Ti/SF/MOF and Ti/SF/I, respectively; **, ## and ++ represent P < 0.01)

TRAP staining was employed to determine osteoclast formation in the BMMs. Figure 4a, d, e revealed that the fewest numbers and areas of osteoclasts were observed in the Ti/SF/MOF/I group, followed by the Ti/SF/I and Ti/SF/MOF groups. We also tested the maturation and function of osteoclasts by bone resorption and F-actin ring staining. Consistent with the TRAP staining results, similar trends were observed in the bone resorption test and F-actin staining: the lowest area of bone resorption and the lowest number of osteoclasts with more than three nuclei were detected in the Ti/SF/MOF/I group (Fig. 4b, c, f, g).

RT-qPCR tests were also carried out to further determine the expression of osteoclastic genes, i.e., Acp5, c-Fos, Oscar, and Sema, at day 5. The results were shown in Fig. 4h–k. BMMs in the Ti/SF/MOF/I group expressed the lowest levels of the selected genes. In summary, the conditioned medium of Ti/SF/MOF/I exerted the strongest effects on inhibiting osteoclast differentiation, which in turn suggested that the Raw264.7 cells cultured on Ti/SF/MOF/I showed the optimal immune regulating effect of inhibiting osteoclast differentiation.

### In vivo mouse air pouch model

**Fig. 5:**
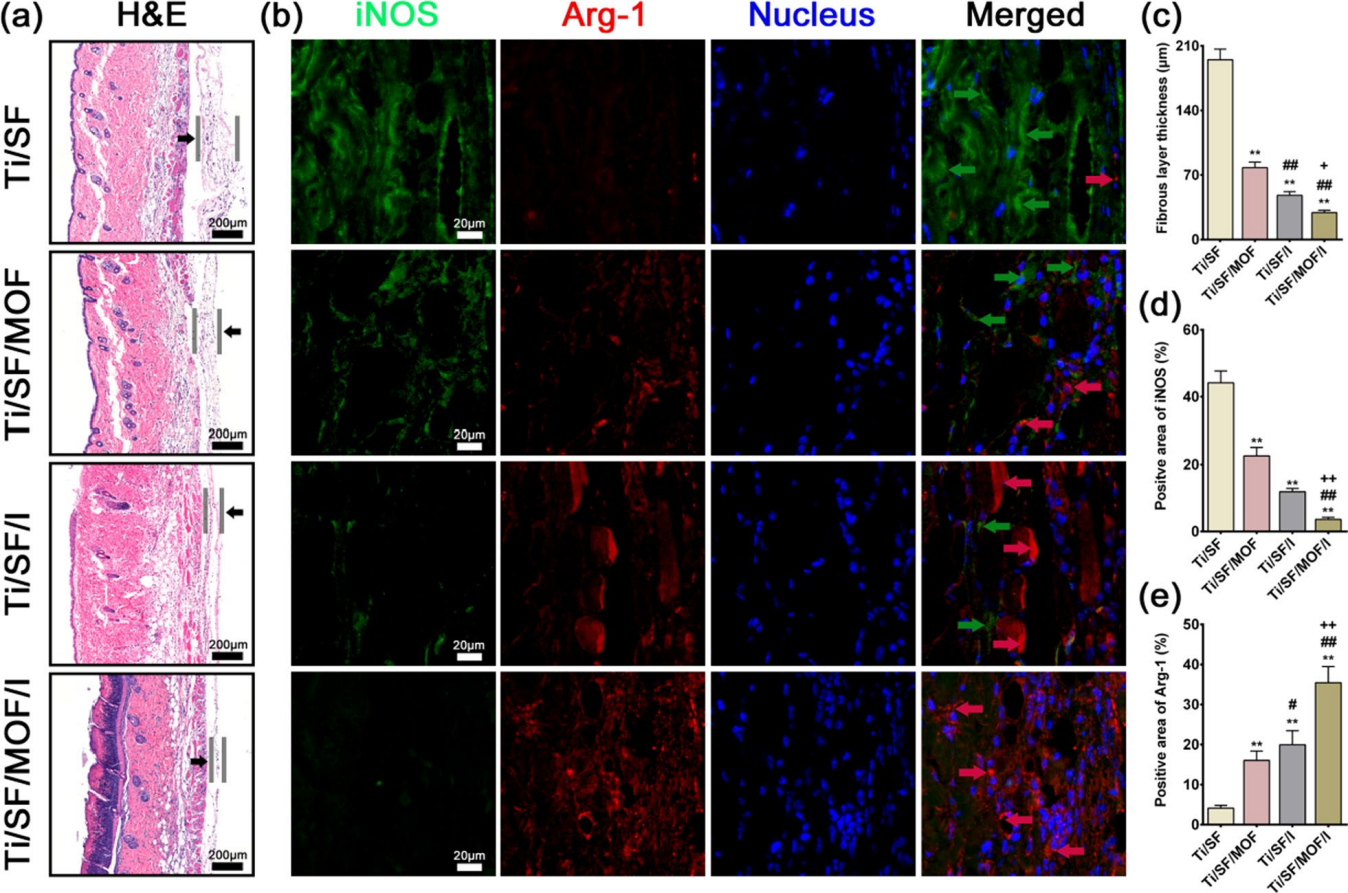
In vivo immunomodulatory evaluation. **a** H&E staining of the air pouches’ skin. **b** Immunofluorescent staining of air pouches’ skin: green (iNOS), red (Arg-1), and blue (Nucleus). **c** Fibrous layer thickness of air pouches’ skin. **d**, **e** Quantitative analysis of iNOS and Arg-1 in immunofluorescent staining. (n = 3; *, # and + represent P < 0.05 when compared with Ti/SF, Ti/SF/MOF and Ti/SF/I, respectively; **, ## and ++ represent P < 0.01)

To evaluate the inflammatory level and different phenotypes of the macrophages infiltrating the air pouch skin, we performed H&E and immunofluorescence staining of the skin sections. The thinnest fibrous layer was observed in the Ti/SF/MOF/I group, which indicated a milder inflammatory reaction than the Ti/SF, Ti/SF/I or Ti/SF/MOF groups (Fig. 5a, c). Further immunofluorescence staining of the fibrous layer suggested that a thicker fibrous layer exhibited more iNOS expression (Ti/SF group, Fig. 5b, d) than a thinner, less inflamed layer, which featured a higher proportion of Arg-1 expression (Ti/SF/MOF/I group, Fig. 5b, e). Therefore, the mouse air pouch model results were consistent with the results of the in vitro experiments, and Ti/SF/MOF/I can induce M2 macrophage switching and bring about an anti-inflammatory environment.

### In vivo osteoporotic bone integration model

**Fig. 6:**
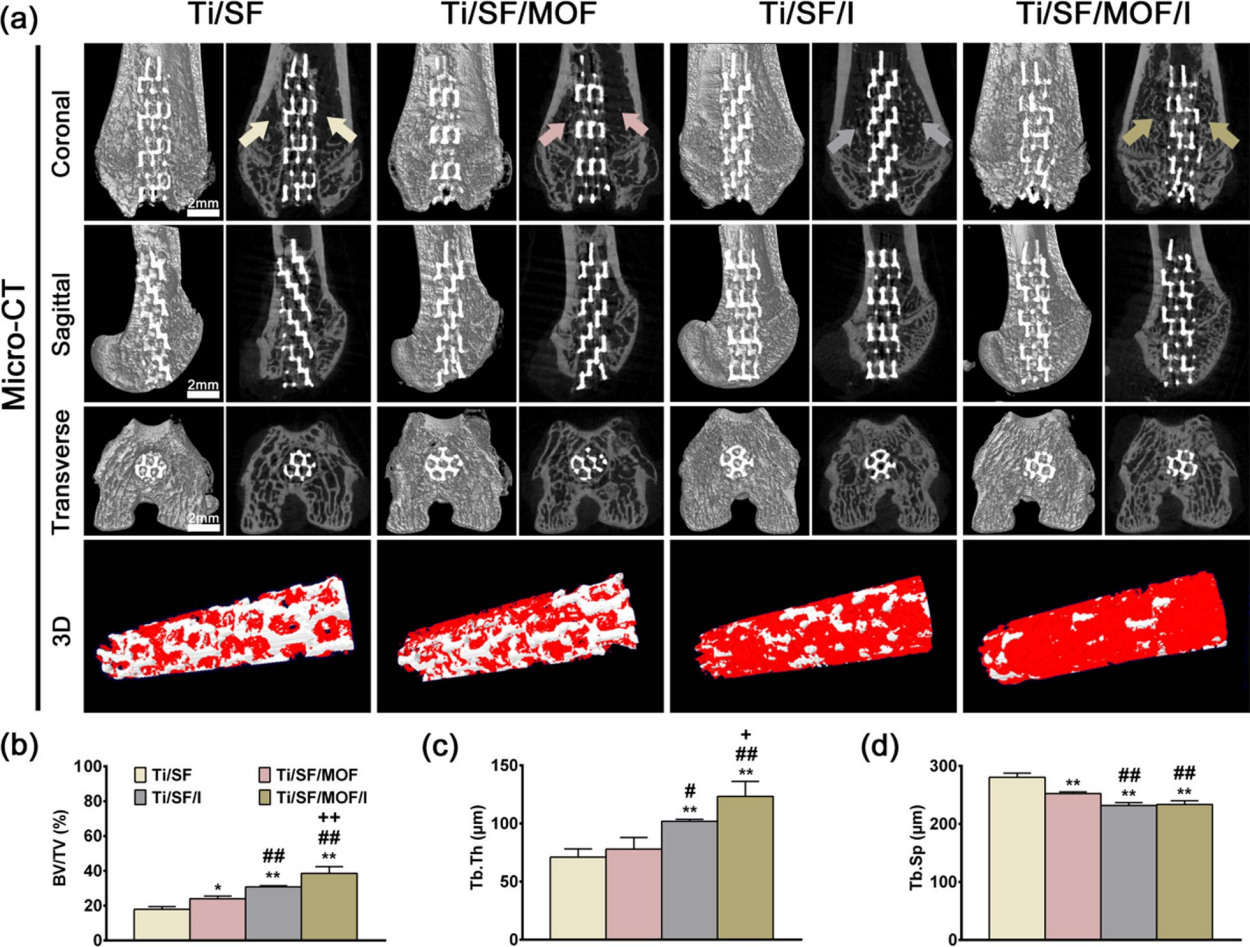
Micro-CT evaluation of osteoporotic osseointegration. **a** Coronal, sagittal, transverse, and 3D images of Micro-CT, red in 3D indicates new bone. **b**–**d** Quantitative analysis of Micro-CT data: BV/TV, Tb. Th, and Tb. Sp respectively. (n = 3; *, # and + represent P < 0.05 when compared with Ti/SF, Ti/SF/MOF and Ti/SF/I, respectively; **, ## and ++ represent P < 0.01)

**Fig. 7:**
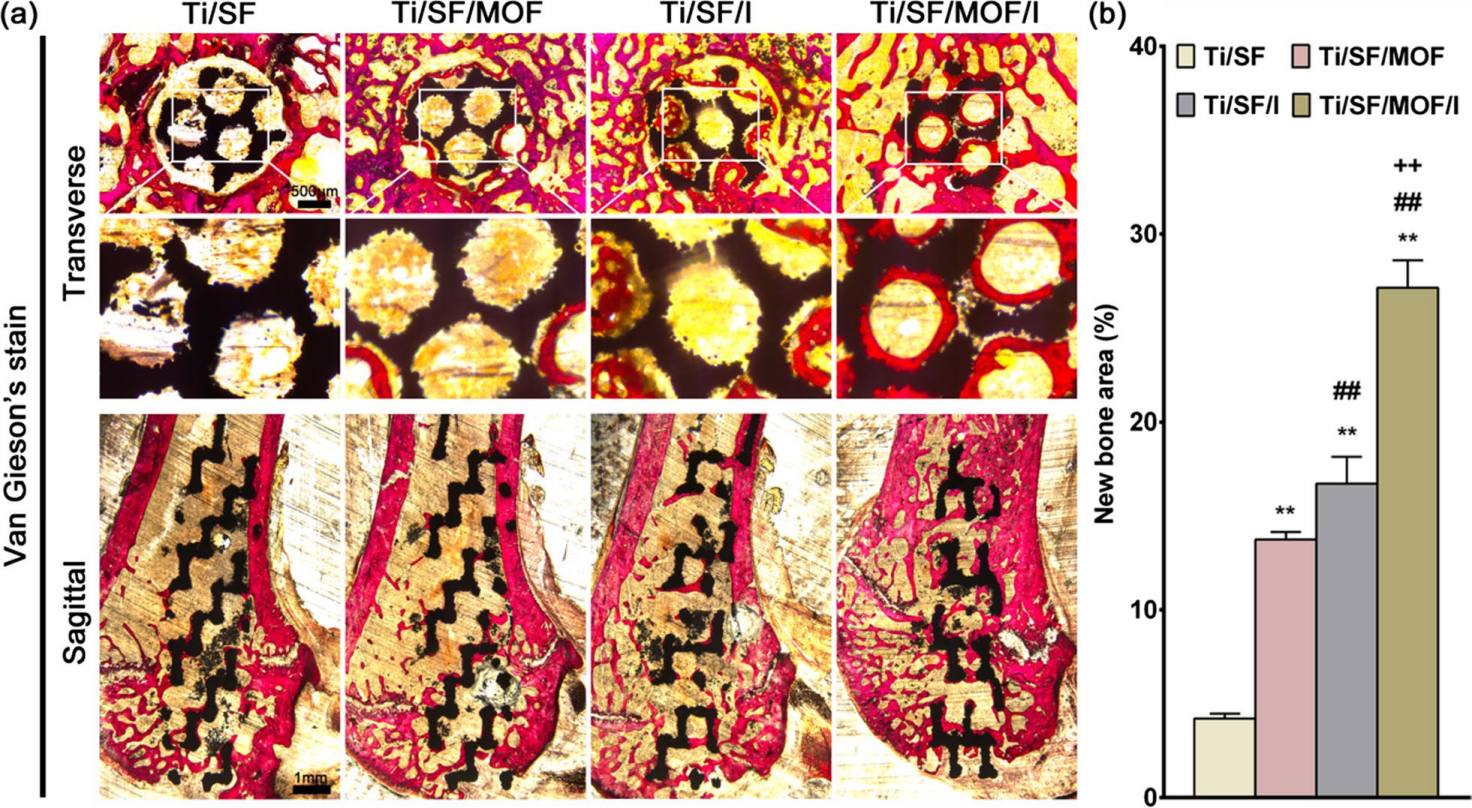
The staining of undecalcified sections. **a** Van Gieson’s staining of undecalcified sections, black indicates implant. **b** Quantitative analysis of van Gieson’s staining data. (n = 3; **, ## and ++ represent P < 0.01 when compared with Ti/SF, Ti/SF/MOF and Ti/SF/I, respectively)

**Fig. 8:**
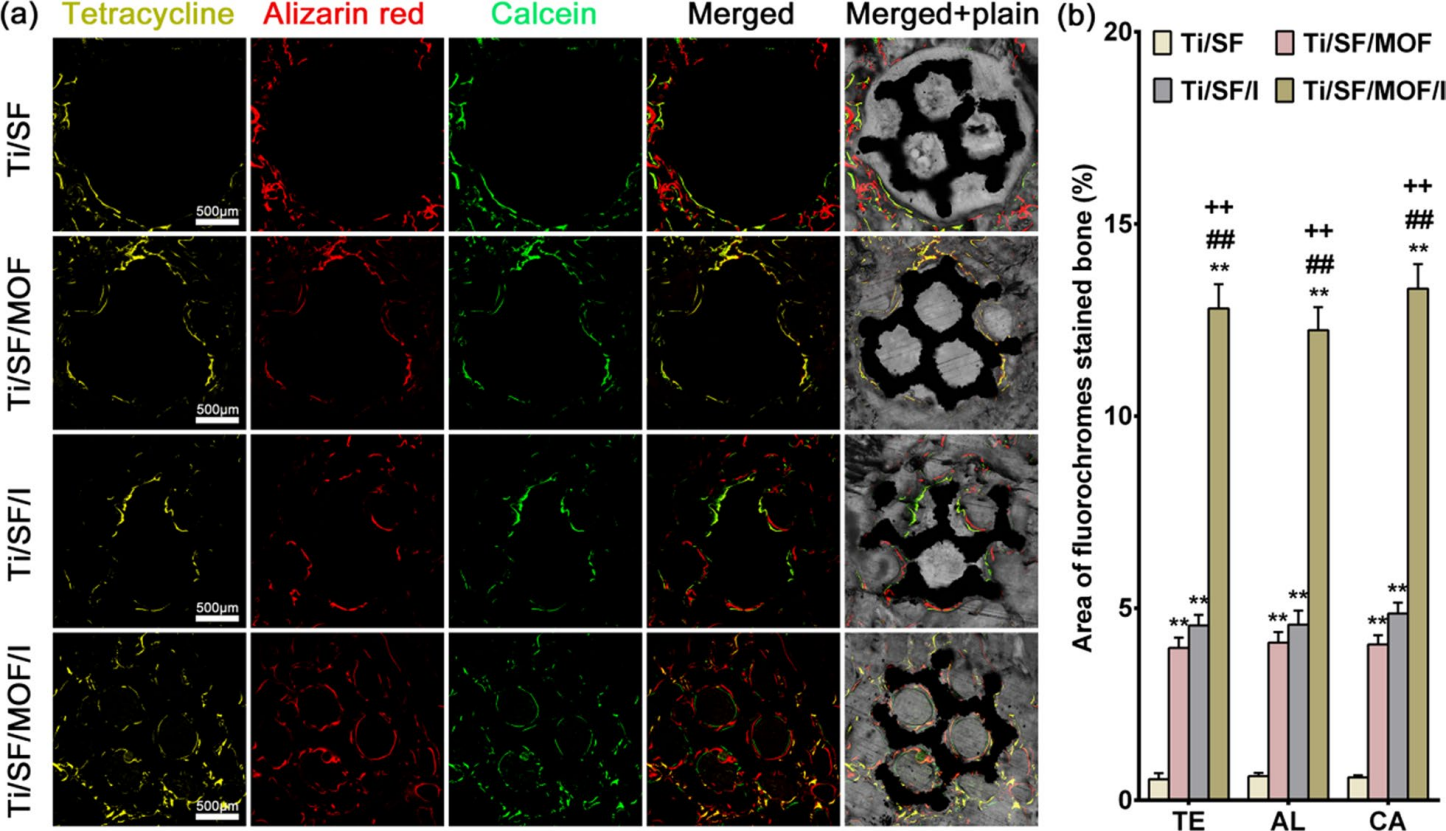
Sequential polychrome labels for bone. **a** Undecalcified sections of sequential polychrome labels for bone: yellow (Tetracycline), red (Alizarin red), and green (Calcein). **b** Quantitative analysis of sequential polychrome labels data. (n = 3; **, ## and ++ represent P < 0.01 when compared with Ti/SF, Ti/SF/MOF and Ti/SF/I, respectively)

Three months after bilateral ovariectomy in female rats, the bone trabecular structure of distal femur decreased significantly, which proved that the model of osteoporosis was successful (Additional file 1: Fig. S5). Figure 6a was the coronal image of the distal femur, which clearly showed the disappearance of the proximal bone trabecular structure of the metaphysis in the Ti/SF and Ti/SF/MOF groups. Moreover, the coronal, sagittal, transverse, and 3D micro-CT images all supported the conclusion that more new bone formed around and inside Ti/SF/MOF/I (Fig. 6a). Further quantitative analysis of the micro-CT data confirmed that the two indexes reflecting new bone formation (BV/TV and Tb. Th) in the Ti/SF/MOF/I group were higher than those in the other groups (Fig. 6b, c), and the Tb. Sp index values reflecting osteoporosis in the Ti/SF/MOF/I and Ti/SF/I groups was lower than those in the Ti/SF and Ti/SF/MOF groups (Fig. 6d). We prepared undecalcified sections to observe osteoporosis and the new bone around and inside the implants through van Gieson and sequential fluorescent labelling staining. Figure 7a revealed that the porous scaffold of Ti/SF/MOF/I was filled with a large amount of new bone; in contrast, there was little new bone in the Ti/SF group, and the bone trabecular structure around the scaffold disappeared. Quantitative analysis indicated that the highest amount of new bone in contact with the implant was in the Ti/SF/MOF/I group, followed by the Ti/SF/I group (Fig. 7b). Sequential fluorescent labelling staining was applied to mark new bone formation around the scaffolds at different time points (Fig. 8a). The naked eye revealed that the highest new bone formation under the pathological condition of osteoporosis at three different time points was observed in the Ti/SF/MOF/I group. Quantitative analysis further indicated that the highest new bone-implant contact rate was in the Ti/SF/MOF/I group, followed by the Ti/SF/I group (Fig. 8b), suggesting that Ti/SF/MOF/I had the strongest osseointegration ability under osteoporosis.

## Discussion

The treatment of osteoporotic prosthesis loosening is a difficult clinical problem [1], and the key to its solution is to achieve bone ingrowth on the surface of implants under the condition of osteoporosis. Osteoporotic patients are particularly prone to implant loosening due to increased bone resorption, especially in older women with postmenopausal oestrogen deficiency [39]. An increasing number of studies have attempted to solve this problem by building sustained-release systems on the surface of implants. However, due to the biological inertness of PT, it is difficult to build a sustained-release system on the surface or inside of PT, so the results of related studies are not as good as expected, especially in the pathological condition of osteoporosis. In addition, the field of bone immunology has been widely studied in recent years. To achieve a balance between bone immunity and bone integration, many strategies have been applied to modify biomaterials to modulate the associated immune response. Recently, some studies have shown that modified biomaterials can modulate the immune system to improve osteogenesis and promote osteointegration [36]. Specifically, it has been reported that cytokines or drug-loaded biomaterials can utilize macrophages to polarize and generate an osteogenic immune microenvironment [40]. However, osseointegration is not only related to osteogenesis but also closely related to bone resorption, especially in patients with osteoporosis. Macrophages are an important component of innate immunity and a primary source of osteoclast progenitor cells. Thus, in addition to the study of macrophage-related osteoblast differentiation, it is necessary to study macrophage-related bone resorption. Therefore, on the basis of the bionic ECM PT biofunctionalization method, this study proposes a biofunctionalization method based on ICA@MOF drug carrier forming a composite bionic ECM to solve osteoporosis prosthesis loosening and studied its possible immune pathway to promote osteoporotic osseointegration.

Clinically, some prosthesis loosening is caused by the stress shielding effect, which can be prevented by a low elastic modulus [41]. The elastic modulus of Ti/SF/MOF/I is lower than that of human cortical bone (5.44 ± 1.25 GPa) in our study [42]. According to the findings proposed by Pobloth et al. and Reznikov et al., a low elastic modulus can enhance bone regeneration into the PT [8, 43]. Moreover, the yield strength of the samples is within the range of human cortical bone (33–193 MPa) [44]. In addition, Barba et al. proposed that the preferred pore size range for bone colonization and bone vascularization is 300–600 μm [45]. Porosity is also an important structural parameter for bone regeneration [46]. According to the previous literature, a porosity of 75% is favourable for bone tissue ingrowth [47]. As shown by our Additional file 1: Fig. S6, PT implants show good bone integration effects in nonosteoporotic conditions. Therefore, PT provides suitable structural and biomechanical properties for improving osteointegration.

Compared with systemic medication, loading drugs in the internal sustained-release carrier of the implant can enable the slow release of the drugs into the microenvironment around the implant, which can not only reduce drug dosage and complications but also achieve better therapeutic effects [48]. Based on the above ideas, this study proposed a drug sustained-release system for osteoporosis treatment with Mg-MOF-74 as the drug carrier, and icariin was loaded to obtain ICA@MOF. The loading method proposed in this study can realize the stable adsorption of icariin by MOF. The biofunctionalized PT Ti/SF/MOF/I was obtained by mixing ICA@MOF and SF into PT and freeze-drying. The pore size of the SF network is larger than 100 μm, so the network will not impede cell immigration and tissue ingrowth [5]. The SF 3D network can provide more adhesion sites for cell attachment and proliferation [49]. In addition, SF can be digested by protease in body fluids, and the degradation products are amino acids and can be used for cell metabolism [50, 51]. Therefore, the biodegradable SF network can provide mechanical support for cell adhesion, and its degradation products are nutrients for cell growth, which can stimulate bone tissue ingrowth. In this composite sustained-release system, MOF will decompose in a liquid environment, especially in an acidic liquid environment [25]. The SF coated on the MOF improved its stability in body fluid, especially in the surgical position (usually a pH value < 7 in the microenvironment). Because SF is biodegradable, MOFs are expected to be gradually exposed to body fluids with the enzymatic hydrolysis of protease in the body fluid, thereby avoiding the burst release phenomenon, so that the scaffold could release icariin stably and effectively for a long time, achieving a better therapeutic effect than simple SF drug loading. Moreover, Mg^2+^ has been proven to be a fundamental element for regulating bone cells and macrophages and can actively participate in the bone tissue regeneration process [20, 52]. The results of our in vivo experiment under osteoporosis conditions are consistent with those reported in the literature. Although there may be concerns about the biosafety of MOF nanomaterials, a number of recent studies have shown that low concentrations of MOF nanomaterials have no significant biotoxicity [53, 54].

Although icariin and Mg^2+^ play an immunomodulatory role in the immune system, the immunomodulatory effects of icariin and Mg^2+^ and their influence on osteogenic and osteoclastic processes are still unclear [55, 56]. In this study, when Raw264.7 cells were inoculated with the sample, some of the macrophages were activated due to the release of icariin and Mg^2+^ into the medium. Most activated macrophages in the Ti/SF group were activated from the M0 phenotype to the M1 phenotype, and a very small portion of activated macrophages adopted the M2 phenotype (Fig. 2h). In contrast, M2 macrophages were predominant in the Ti/SF/I and Ti/SF/MOF/I groups, especially in the Ti/SF/MOF/I group. In summary, in vitro experiments have shown that Ti/SF/MOF/I can facilitate the polarization of macrophages to the M2 phenotype and the secretion of anti-inflammatory factors (Fig. 2). In fact, some previous reports have suggested that icariin has a regulatory effect on the immune system. Specifically, icariin induces the differentiation of monocytes into macrophages, and 7.5–30 μM icariin significantly inhibits the release of proinflammatory cytokines such as IL-6 and TNF-α in a dose-dependent manner [55, 56]. The results of the release tests of Ti/SF/MOF/I and Ti/SF/I showed that the concentration of icariin were approximately 7.39 μM and 23.65 μM, respectively. However, it should be noted that the actual icariin concentration on the surface of the material with which the cells interact may be higher. Therefore, the optimal concentration of icariin in the MOF needs to be further studied to avoid cytotoxic side effects and waste of resources. In addition, other studies have shown that icariin regulates cytokine secretion in macrophages by inhibiting NF-κB signaling [56]. It is well known that inhibition of NF-κB signaling in macrophages leads M0 macrophages to convert to the anti-inflammatory M2 phenotype. Further analysis of the mechanism confirmed that icariin can inhibit NF-κB signaling through the upregulation of the PI3K/Akt pathway and reduce the production of IL-6 and TNF-α [56]. Our ELISA and RT-qPCR results were consistent with the results of previous studies. It has been confirmed that Mg^2+^ promotes osteogenesis via anti-inflammatory immunoregulation, but the optimal concentration of Mg^2+^ is still unknown [57, 58]. In our study, immunofluorescence staining and ELISA results confirmed that the intensity of M2 macrophages in the Ti/SF/MOF/I group was higher than that in the Ti/SF/I group (Fig. 2), which was mainly attributed to the release of a certain concentration of Mg^2+^ in the MOF. This further explained the high concentration of icariin in Ti/SF/I group, but its anti-inflammatory effect is weaker than that of Ti/SF/MOF/I group. Compared with Ti/SF/I and Ti/SF/MOF, Ti/SF/MOF/I had the highest intensity of M2 macrophages, mainly due to the synergistic immune regulation of the SF/ICA@MOF sustained-release system. In addition, more and more studies have reported that the Notch1 pathway plays an important role in promoting inflammatory macrophage polarization [59, 60]. In our study, we also found that a certain concentration of icariin and Mg^2+^ could significantly reduce the expression of Notch1 protein. These results suggest that ICA@MOF may play a biological role in promoting M0-type macrophage polarization to M2-type through synergistic inhibition of Notch1 pathway.

Acute and uncontrolled inflammation can lead to implant failure in vivo [29]. In contrast, a mild immune response of biological materials is conducive to the stability of the implant and bone integration [36]. Therefore, to evaluate the ability of the Ti/SF/MOF/I-generated immune microenvironment to regulate both osteogenesis and osteoclast differentiation, we cultured rBMSCs and BMMs in macrophage-conditioned medium. Ti/SF/MOF/I showed the strongest osteogenic differentiation and osteoclast differentiation inhibition (Figs. 3, 4), indicating that macrophages (M0) cultured on Ti/SF/MOF/I had the highest potential osteoblast-promoting and osteoclast-inhibiting molecular secretion. It is well known that pro-inflammatory cytokines, especially TNF, can significantly inhibit osteogenic differentiation by inhibiting BMP pathway and enhance RANKL pathway to promote osteoclast differentiation [61, 62]. In our study, Ti/SF/MOF/I significantly inhibited the secretion of pro-inflammatory factors, especially TNF-α, which was similar to the results of previous studies.

Previous in vitro experiments have demonstrated that sustained-release system of PT-delivered SF/ICA@MOF can induce macrophages to polarize into the M2 phenotype, thereby promoting osteogenic differentiation and inhibiting osteoclast differentiation. Then, we further investigated the in vivo inflammatory response and macrophage polarization using a mouse balloon model. A thinner fibrous layer was observed in the Ti/SF/MOF/I group, indicating a relative anti-inflammatory effect (Fig. 5a, c). In addition, immunofluorescence images (Fig. 5b, d, e) showed a higher intensity of M2 macrophages, which may be the reason for the lower inflammation in the Ti/SF/MOF/I group. When the sample is implanted into a balloon, the foreign body reaction is activated, and neutrophils and macrophages are the first reactive cells to cluster around the implant [63]. For Ti/SF/MOF/I, a higher proportion of macrophages were induced to an M2 phenotype, resulting in the secretion of anti-inflammatory cytokines (such as IL-4 and IL-10). In addition, M2 macrophages produce less IL-6 and TNF-α, thus maintaining a milder inflammatory microenvironment. To verify whether the in vitro osteogenic and osteoclast effects were consistent with the in vivo results, we further evaluated the osteointegration ability of Ti/SF/MOF/I using an osteoporotic bone defect repair model.

In this study, Ti/SF/MOF/I showed good effects on bone integration, indicating that Ti/SF/MOF/I is suitable for bone integration under the conditions of osteoporosis. Biofunctionalization is expected to improve the long-term stability of PT in patients with osteoporosis, especially elderly patients who need prosthesis replacement. In addition, an interesting phenomenon was noted in the results. After the implantation of PT, only the sequence fluorescence images of Ti/SF/MOF/I scaffolds were highly inconsistent at three time points, which indicated that new bone was continuously generated in this group of samples. Although Ti/SF/I and Ti/SF/MOF scaffolds had a certain degree of bone insertion, the vast majority of new bone formation occurred only in the first 2 weeks, and bone remodelling was only carried out on the basis of the new bone in the later period. This result further proves that SF/ICA@MOF sustained-release carriers can realize the long-term sustained release of icariin in vivo, change the local microenvironment, and tilt local bone metabolism in the direction of osteogenesis and of osteoclast inhibition within 2 months. Therefore, the biofunctionalized PT proposed in this study can realize the long-term release of icariin and regulate the local immune microenvironment around the PT to achieve good bone integration in osteoporosis, which has important clinical significance.

## Conclusion

In summary, we developed a hierarchical biofunctionalized PT implant accompanied with controllable release of icariin and Mg^2+^ in the microenvironment. In this hierarchical biofunctionalized PT implant, the macro pores provide mechanical support, the ECM-like structure provides cellular support and the embedded nanoparticles exert biological effects. In vitro and in vivo experiments demonstrated that the hierarchical biofunctionalized PT significantly improved osteoporotic integration by inducing M0 macrophages to M2-type and secreted anti-inflammatory factors through sustained release of icariin and Mg^2+^. Our results showed that the hierarchical biofunctionalized PT implant can regulate bone metabolism through immune pathway and achieve enhanced osteointegration between the implant and host osteoporotic bone, which provides a strategy for the optimization of titanium alloy femur prosthesis in elderly patients with osteoporosis.

## Supplementary Information

**Additional file 1: Table S1.** Primers used for RT-qPCR of Raw264.7 cells. **Table S2. **Primers used for RT-qPCR of rBMSC cells. **Table S3.** Primers used for RT-qPCR of BMM cells. **Figure S1.** Mechanical properties of different samples. **Figure S2.** The biocompatibility of scaffolds extracts to Raw264.7 cells was detected by live and death staining, green represents living cells and red represents dead cells. **Figure S3. **The expressions of osteogenic differentiation genes in rBMSCs were detected by RT-qPCR. (*, # and + represent P < 0.05 when compared with Ti/SF, Ti/SF/MOF and Ti/SF/I, respectively; **, ## and ++ represent P < 0.01). **Figure S4. **The expressions of RUNX2 in rBMSCs were detected by western blotting. (*, # and + represent P < 0.05 when compared with Ti/SF, Ti/SF/MOF and Ti/SF/I, respectively; **, ## and ++ represent P < 0.01). **Figure S5.** The bone mass of femoral condyle in female SD rats was measured by Micro-CT after 12 weeks of bilateral ovariectomy. **Figure S6. **Van Gieson staining of undecalcified sections after samples implantation 8 weeks in normal female SD rats.

## Data Availability

The data used or analysed during the current study are available from the corresponding author on reasonable request.

## References

[1] Cherubino P, Ratti C, Fagetti A, Binda T (2011). Total hip arthroplasty and bone fragility. Aging Clin Exp Res.

[2] Alexiou KI, Roushias A, Varitimidis SE, Malizos KN (2018). Quality of life and psychological consequences in elderly patients after a hip fracture: a review. Clin Interv Aging.

[3] Wan KH, Choi ST, Wong KK, Wong KK (2020). Nightmare of repeated low-energy periprosthetic femoral fracture in a patient with severe osteoporosis—a lesson learned. Trauma Case Rep.

[4] Rivière C, Grappiolo G, Engh CA, Vidalain JP, Chen AF, Boehler N, Matta J, Vendittoli PA (2018). Long-term bone remodelling around 'legendary' cementless femoral stems. EFORT Open Rev.

[5] Wang X, Xu S, Zhou S, Xu W, Leary M, Choong P, Qian M, Brandt M, Xie YM (2016). Topological design and additive manufacturing of porous metals for bone scaffolds and orthopaedic implants: a review. Biomaterials.

[6] Zadpoor AA (2019). Additively manufactured porous metallic biomaterials. J Mater Chem B.

[7] Charbonnier B, Hadida M, Marchat D (2021). Additive manufacturing pertaining to bone: hopes, reality and future challenges for clinical applications. Acta Biomater.

[8] Pobloth AM, Checa S, Razi H, Petersen A, Weaver JC, Schmidt-Bleek K, Windolf M, Tatai A, Roth CP, Schaser KD (2018). Mechanobiologically optimized 3D titanium-mesh scaffolds enhance bone regeneration in critical segmental defects in sheep. Sci Transl Med.

[9] Li Z, Müller R, Ruffoni D (2018). Bone remodeling and mechanobiology around implants: insights from small animal imaging. J Orthop Res.

[10] Kyllönen L, D'Este M, Alini M, Eglin D (2015). Local drug delivery for enhancing fracture healing in osteoporotic bone. Acta Biomater.

[11] Tabatabaei-Malazy O, Salari P, Khashayar P, Larijani B (2017). New horizons in treatment of osteoporosis. Daru.

[12] Wang Z, Wang D, Yang D, Zhen W, Zhang J, Peng S (2018). The effect of icariin on bone metabolism and its potential clinical application. Osteoporos Int.

[13] Yu T, Xiong Y, Luu S, You X, Li B, Xia J, Zhu H, Zhao Y, Zhou H, Yu G, Yang Y (2020). The shared KEGG pathways between icariin-targeted genes and osteoporosis. Aging (Albany NY).

[14] Zadpoor AA (2019). Meta-biomaterials. Biomater Sci.

[15] Izquierdo-Barba I, Santos-Ruiz L, Becerra J, Feito MJ, Fernández-Villa D, Serrano MC, Díaz-Güemes I, Fernández-Tomé B, Enciso S, Sánchez-Margallo FM (2019). Synergistic effect of Si-hydroxyapatite coating and VEGF adsorption on Ti6Al4V-ELI scaffolds for bone regeneration in an osteoporotic bone environment. Acta Biomater.

[16] Davoodi E, Montazerian H, Esmaeilizadeh R, Darabi AC, Rashidi A, Kadkhodapour J, Jahed H, Hoorfar M, Milani AS, Weiss PS (2021). Additively manufactured gradient porous Ti-6Al-4V hip replacement implants embedded with cell-laden gelatin methacryloyl hydrogels. ACS Appl Mater Interfaces.

[17] Yavari SA, Croes M, Akhavan B, Jahanmard F, Eigenhuis CC, Dadbakhsh S, Vogely HC, Bilek MM, Fluit AC, Boel CHE (2020). Layer by layer coating for bio-functionalization of additively manufactured meta-biomaterials. Addit Manuf.

[18] Kenkre JS, Bassett J (2018). The bone remodelling cycle. Ann Clin Biochem.

[19] Mohammadi M, Mousavi Shaegh SA, Alibolandi M, Ebrahimzadeh MH, Tamayol A, Jaafari MR, Ramezani M (2018). Micro and nanotechnologies for bone regeneration: recent advances and emerging designs. J Control Release.

[20] Behzadi S, Luther GA, Harris MB, Farokhzad OC, Mahmoudi M (2017). Nanomedicine for safe healing of bone trauma: opportunities and challenges. Biomaterials.

[21] Li Z, Zhang X, Ouyang J, Chu D, Han F, Shi L, Liu R, Guo Z, Gu GX, Tao W (2021). Ca(2+)-supplying black phosphorus-based scaffolds fabricated with microfluidic technology for osteogenesis. Bioact Mater.

[22] Wu MX, Yang YW (2017). Metal-organic framework (MOF)-based drug/cargo delivery and cancer therapy. Adv Mater.

[23] Horcajada P, Gref R, Baati T, Allan PK, Maurin G, Couvreur P, Férey G, Morris RE, Serre C (2012). Metal-organic frameworks in biomedicine. Chem Rev.

[24] Hu Q, Yu J, Liu M, Liu A, Dou Z, Yang Y (2014). A low cytotoxic cationic metal-organic framework carrier for controllable drug release. J Med Chem.

[25] Shen X, Zhang Y, Ma P, Sutrisno L, Luo Z, Hu Y, Yu Y, Tao B, Li C, Cai K (2019). Fabrication of magnesium/zinc-metal organic framework on titanium implants to inhibit bacterial infection and promote bone regeneration. Biomaterials.

[26] Li X, Xiong YZ, Zhang H, Gao RN (2021). Development of functionally graded porous titanium/silk fibroin composite scaffold for bone repair. Mater Lett.

[27] Thurber AE, Omenetto FG, Kaplan DL (2015). In vivo bioresponses to silk proteins. Biomaterials.

[28] Wenk E, Merkle HP, Meinel L (2011). Silk fibroin as a vehicle for drug delivery applications. J Control Release.

[29] Bai J, Wang H, Chen H, Ge G, Wang M, Gao A, Tong L, Xu Y, Yang H, Pan G (2020). Biomimetic osteogenic peptide with mussel adhesion and osteoimmunomodulatory functions to ameliorate interfacial osseointegration under chronic inflammation. Biomaterials.

[30] Li L, Yu M, Li Y, Li Q, Yang H, Zheng M, Han Y, Lu D, Lu S, Gui L (2021). Synergistic anti-inflammatory and osteogenic n-HA/resveratrol/chitosan composite microspheres for osteoporotic bone regeneration. Bioact Mater.

[31] Whitaker R, Hernaez-Estrada B, Hernandez RM, Santos-Vizcaino E, Spiller KL (2021). Immunomodulatory biomaterials for tissue repair. Chem Rev.

[32] Martin KE, García AJ (2021). Macrophage phenotypes in tissue repair and the foreign body response: implications for biomaterial-based regenerative medicine strategies. Acta Biomater.

[33] McDonald MM, Khoo WH, Ng PY, Xiao Y, Zamerli J, Thatcher P, Kyaw W, Pathmanandavel K, Grootveld AK, Moran I (2021). Osteoclasts recycle via osteomorphs during RANKL-stimulated bone resorption. Cell.

[34] Jiang C, Zhou Z, Lin Y, Shan H, Xia W, Yin F, Wang N, Zhou L, Gao Y, Yu X (2021). Astragaloside IV ameliorates steroid-induced osteonecrosis of the femoral head by repolarizing the phenotype of pro-inflammatory macrophages. Int Immunopharmacol.

[35] Wang W, Liu Y, Yang C, Qi X, Li S, Liu C, Li X (2019). Mesoporous bioactive glass combined with graphene oxide scaffolds for bone repair. Int J Biol Sci.

[36] Liu W, Li J, Cheng M, Wang Q, Yeung KWK, Chu PK, Zhang X (2018). Zinc-modified sulfonated polyetheretherketone surface with immunomodulatory function for guiding cell fate and bone regeneration. Adv Sci (Weinh).

[37] Wang W, Liu Y, Yang C, Jia W, Qi X, Liu C, Li X (2020). Delivery of salvianolic acid B for efficient osteogenesis and angiogenesis from silk fibroin combined with graphene oxide. ACS Biomater Sci Eng.

[38] Deng H, Grunder S, Cordova KE, Valente C, Furukawa H, Hmadeh M, Gándara F, Whalley AC, Liu Z, Asahina S (2012). Large-pore apertures in a series of metal-organic frameworks. Science.

[39] Liu J, Curtis EM, Cooper C, Harvey NC (2019). State of the art in osteoporosis risk assessment and treatment. J Endocrinol Invest.

[40] He Y, Yang X, Yuan Z, Shen X, Xu K, Lin C, Tao B, Li K, Chen M, Hu Y (2019). Regulation of MSC and macrophage functions in bone healing by peptide LL-37-loaded silk fibroin nanoparticles on a titanium surface. Biomater Sci.

[41] Liverani E, Rogati G, Pagani S, Brogini S, Fortunato A, Caravaggi P (2021). Mechanical interaction between additive-manufactured metal lattice structures and bone in compression: implications for stress shielding of orthopaedic implants. J Mech Behav Biomed Mater.

[42] Choi K, Kuhn JL, Ciarelli MJ, Goldstein SA (1990). The elastic moduli of human subchondral, trabecular, and cortical bone tissue and the size-dependency of cortical bone modulus. J Biomech.

[43] Reznikov N, Boughton OR, Ghouse S, Weston AE, Collinson L, Blunn GW, Jeffers JRT, Cobb JP, Stevens MM (2019). Individual response variations in scaffold-guided bone regeneration are determined by independent strain- and injury-induced mechanisms. Biomaterials.

[44] Zhang XY, Fang G, Zhou J (2017). Additively manufactured scaffolds for bone tissue engineering and the prediction of their mechanical behavior: a review. Materials (Basel).

[45] Barba D, Alabort E, Reed RC (2019). Synthetic bone: design by additive manufacturing. Acta Biomater.

[46] Karageorgiou V, Kaplan D (2005). Porosity of 3D biomaterial scaffolds and osteogenesis. Biomaterials.

[47] Chen H, Han Q, Wang C, Liu Y, Chen B, Wang J (2020). Porous scaffold design for additive manufacturing in orthopedics: a review. Front Bioeng Biotechnol.

[48] Alghamdi M, Gumbleton M, Newland B (2021). Local delivery to malignant brain tumors: potential biomaterial-based therapeutic/adjuvant strategies. Biomater Sci.

[49] Li X, Ma XY, Feng YF, Ma ZS, Wang J, Ma TC, Qi W, Lei W, Wang L (2015). Osseointegration of chitosan coated porous titanium alloy implant by reactive oxygen species-mediated activation of the PI3K/AKT pathway under diabetic conditions. Biomaterials.

[50] Holland C, Numata K, Rnjak-Kovacina J, Seib FP (2019). The biomedical use of silk: past, present, Future. Adv Healthc Mater.

[51] Bhattacharjee P, Kundu B, Naskar D, Kim HW, Maiti TK, Bhattacharya D, Kundu SC (2017). Silk scaffolds in bone tissue engineering: an overview. Acta Biomater.

[52] O'Neill E, Awale G, Daneshmandi L, Umerah O, Lo KW (2018). The roles of ions on bone regeneration. Drug Discov Today.

[53] Yang P, Tao J, Chen F, Chen Y, He J, Shen K, Zhao P, Li Y (2021). Multienzyme-mimic ultrafine alloyed nanoparticles in metal organic frameworks for enhanced chemodynamic therapy. Small.

[54] Luzuriaga MA, Welch RP, Dharmarwardana M, Benjamin CE, Li S, Shahrivarkevishahi A, Popal S, Tuong LH, Creswell CT, Gassensmith JJ (2019). Enhanced stability and controlled delivery of MOF-encapsulated vaccines and their immunogenic response in vivo. ACS Appl Mater Interfaces.

[55] Sun X, Deng X, Cai W, Li W, Shen Z, Jiang T, Huang J (2018). Icariin inhibits LPS-induced cell inflammatory response by promoting GRα nuclear translocation and upregulating GRα expression. Life Sci.

[56] Xu CQ, Liu BJ, Wu JF, Xu YC, Duan XH, Cao YX, Dong JC (2010). Icariin attenuates LPS-induced acute inflammatory responses: involvement of PI3K/Akt and NF-kappaB signaling pathway. Eur J Pharmacol.

[57] Tan S, Wang Y, Du Y, Xiao Y, Zhang S (2021). Injectable bone cement with magnesium-containing microspheres enhances osteogenesis via anti-inflammatory immunoregulation. Bioact Mater.

[58] Lin Z, Shen D, Zhou W, Zheng Y, Kong T, Liu X, Wu S, Chu PK, Zhao Y, Wu J (2021). Regulation of extracellular bioactive cations in bone tissue microenvironment induces favorable osteoimmune conditions to accelerate in situ bone regeneration. Bioact Mater.

[59] Nakano T, Katsuki S, Chen M, Decano JL, Halu A, Lee LH, Pestana DVS, Kum AST, Kuromoto RK, Golden WS (2019). Uremic toxin indoxyl sulfate promotes proinflammatory macrophage activation via the interplay of OATP2B1 and Dll4-notch signaling. Circulation.

[60] Kim MJ, Park JS, Lee SJ, Jang J, Park JS, Back SH, Bahn G, Park JH, Kang YM, Kim SH (2015). Notch1 targeting siRNA delivery nanoparticles for rheumatoid arthritis therapy. J Control Release.

[61] Mukai T, Otsuka F, Otani H, Yamashita M, Takasugi K, Inagaki K, Yamamura M, Makino H (2007). TNF-alpha inhibits BMP-induced osteoblast differentiation through activating SAPK/JNK signaling. Biochem Biophys Res Commun.

[62] Marahleh A, Kitaura H, Ohori F, Kishikawa A, Ogawa S, Shen WR, Qi J, Noguchi T, Nara Y, Mizoguchi I (2019). TNF-α directly enhances osteocyte RANKL expression and promotes osteoclast formation. Front Immunol.

[63] Zhang B, Su Y, Zhou J, Zheng Y, Zhu D (2021). Toward a better regeneration through implant-mediated immunomodulation: harnessing the immune responses. Adv Sci (Weinh).

