# Supplementary material for: A novel hierarchical biofunctionalized 3D-printed porous Ti6Al4V scaffold with enhanced osteoporotic osseointegration through osteoimmunomodulation
Source: J Nanobiotechnology. 2022 Feb 5;20:68. doi: 10.1186/s12951-022-01277-0 (PMC8817481; doi:10.1186/s12951-022-01277-0)
Supplement: Supplementary file 1 — Additional file 1: Table S1. Primers used for RT-qPCR of Raw264.7 cells. Table S2. Primers used for RT-qPCR of rBMSC cells. Table S3. Primers used for RT-qPCR of BMM cells. Figure S1. Mechanical properties of different samples. Figure S2. The biocompatibility of scaffolds extracts to Raw264.7 cells was detected by live and death staining, green represents living cells and red represents dead cells. Figure S3. The expressions of osteogenic differentiation genes in rBMSCs were detected by RT-qPCR. (*, # and + represent P < 0.05 when compared with Ti/SF, Ti/SF/MOF and Ti/SF/I, respectively; **, ## and ++ represent P < 0.01). Figure S4. The expressions of RUNX2 in rBMSCs were detected by western blotting. (*, # and + represent P < 0.05 when compared with Ti/SF, Ti/SF/MOF and Ti/SF/I, respectively; **, ## and ++ represent P < 0.01). Figure S5. The bone mass of femoral condyle in female SD rats was measured by Micro-CT after 12 weeks of bilateral ovariectomy. Figure S6. Van Gieson staining of undecalcified sections after samples implantation 8 weeks in normal female SD rats. [file 12951_2022_1277_MOESM1_ESM.docx]

Additional Information

**A novel hierarchical biofunctionalized 3D-printed porous Ti6Al4V scaffold with enhanced osteoporotic osseointegration through osteoimmunomodulation**

Wei Wang, Yinze Xiong, Renliang Zhao, Xiang Li^*^, Weitao Jia^*^

**Table S1**. Primers used for RT-qPCR of Raw264.7 cells

| Genes | Primers Sequence (F, forward; R, reverse; 5’−3’) |
| --- | --- |
| TNF-α | F: TAGCCAGGAGGGAGAACAGA |
|  | R: CCAGTGAGTGAAAGGGACAGA |
| iNOS | F: TTGACGCTCGGAACTGTA |
|  | R: GTTGGTGGCATAAAGTATGTG |
| Arg-1 | F: TGCTCACACTGACATCAACAC |
|  | R: GAGAATCCTGGTACATCTGGG |
| IL-10 | F: GAGAAGCATGGCCCAGAAATC |
|  | R: GAGAAATCGATGACAGCGCC |

**Table S2.** Primers used for RT-qPCR of rBMSC cells

| Genes | Primers Sequence (F, forward; R, reverse; 5’−3’) |
| --- | --- |
| ALP | F: GGATCAAAGCAGCATCTTACCAG |
|  | R: GCTTTCCCATCTTCCGACACT |
| BMP-2 | F: AACGAGAAAAGCGTCAAGCC |
|  | R: AGGTGCCACGATCCAGTCAT |
| RUNX2 | F: CCTGAACTCAGCACCAAGTCCT |
|  | R: TCAGAGGTGGCAGTGTCATCA |
| OCN | F: CAGACAAGTCCCACACAGCA |
|  | R: CCAGCAGAGTGAGCAGAGAG |

**Table S3.** Primers used for RT-qPCR of BMM cells

| Genes | Primers Sequence (F, forward; R, reverse; 5’−3’) |
| --- | --- |
| TRAP | F: CACTCCCACCCTGAGATTTGT |
|  | R: CCCCAGAGACATGATGAAGTCA |
| c-Fos | F: CCAGTCAAGAGCATCAGCAA |
|  | R: AAGTAGTGCAGCCCGGAGTA |
| OSCAR | F: CTGCTGGTAACGGATCAGCTCCCCAGA |
|  | R: CCAAGGAGCCAGAACCTTCGAAACT |
| SEMA | F: TAAAGTGAATGAAACCATTTGT |
|  | R: GTCTGTGAAATGTTTTACAGTGT |


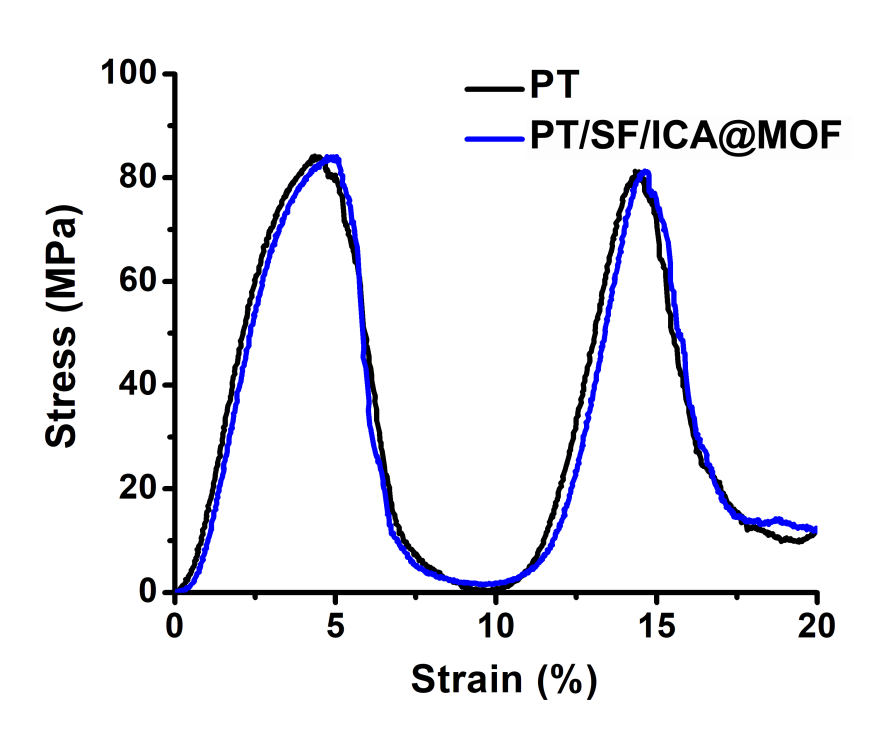


**Figure S1.** Mechanical properties of different samples.


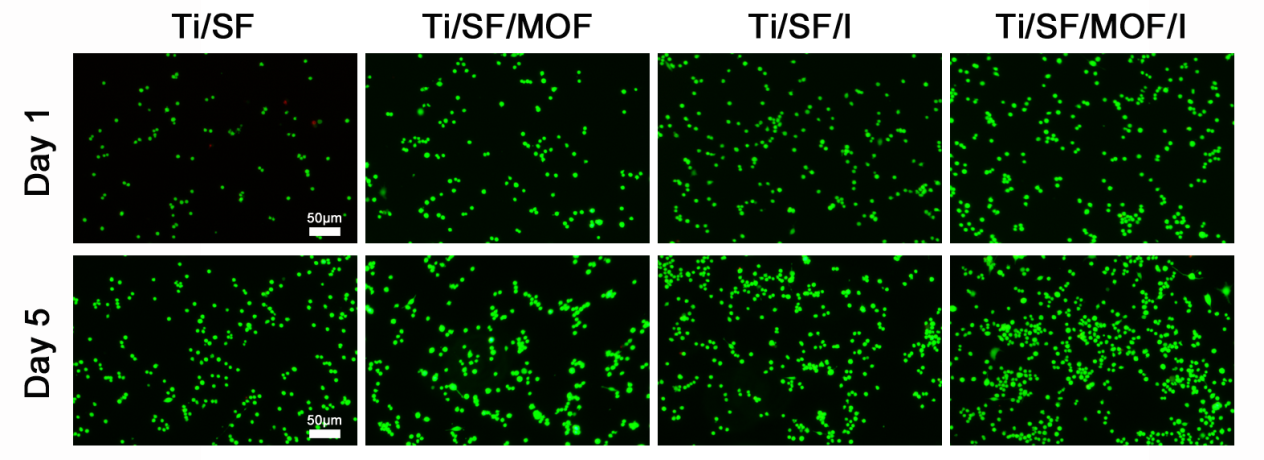


**Figure S2.** The biocompatibility of scaffolds extracts to Raw264.7 cells was detected by live and death staining, green represents living cells and red represents dead cells.


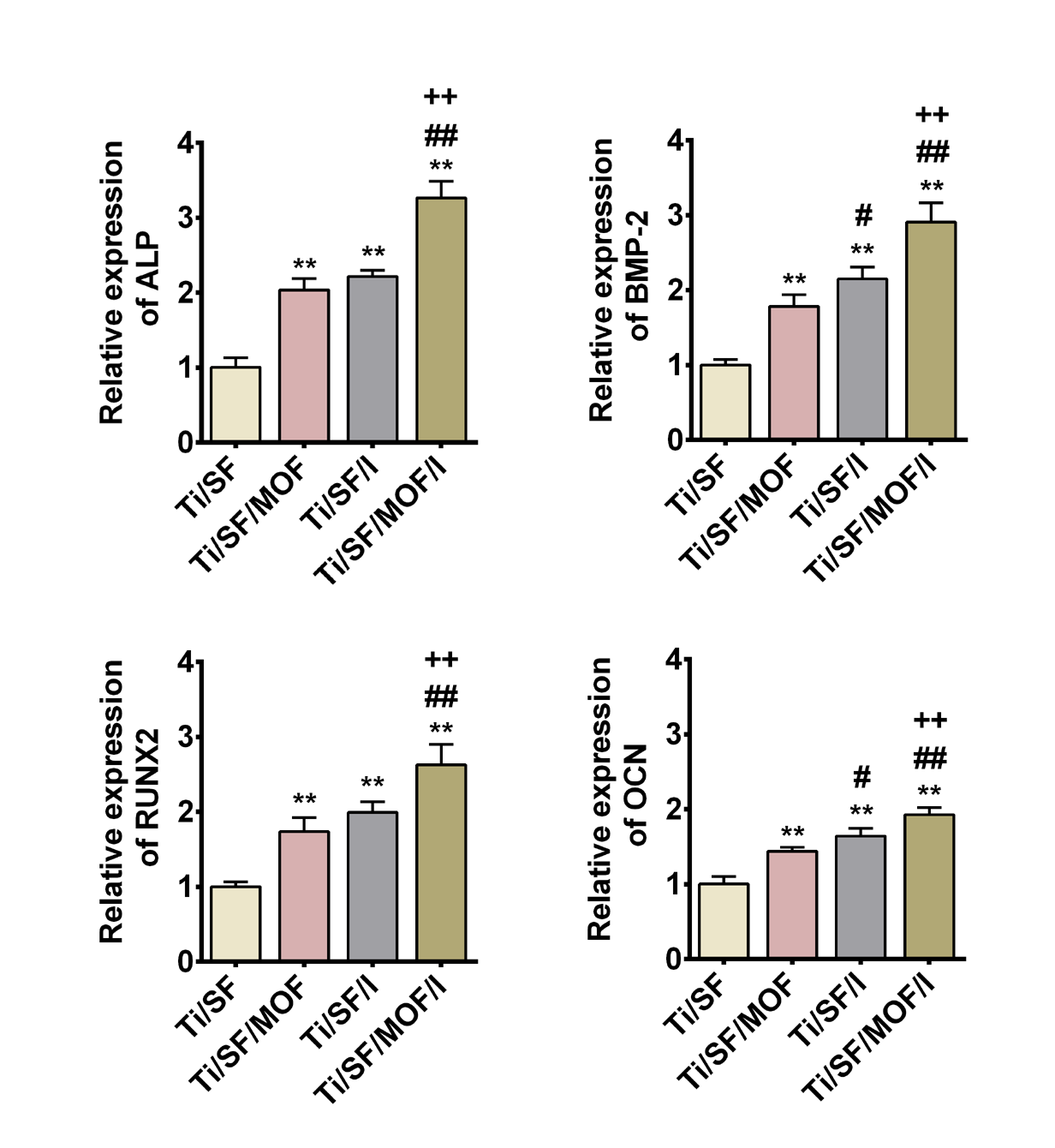


**Figure S3.** The expressions of osteogenic differentiation genes in rBMSCs were detected by RT-qPCR. (*, # and + represent P < 0.05 when compared with Ti/SF, Ti/SF/MOF and Ti/SF/I, respectively; **, ## and ++ represent P < 0.01)


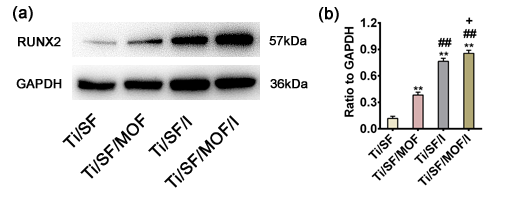


**Figure S4.** The expressions of RUNX2 in rBMSCs were detected by western blotting. (*, # and + represent P < 0.05 when compared with Ti/SF, Ti/SF/MOF and Ti/SF/I, respectively; **, ## and ++ represent P < 0.01)


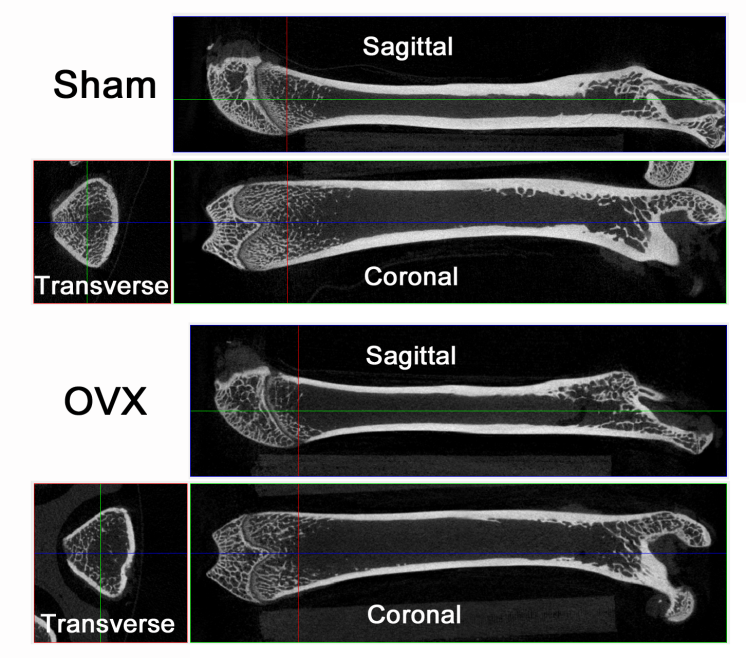


**Figure S5.** The bone mass of femoral condyle in female SD rats was measured by Micro-CT after 12 weeks of bilateral ovariectomy.


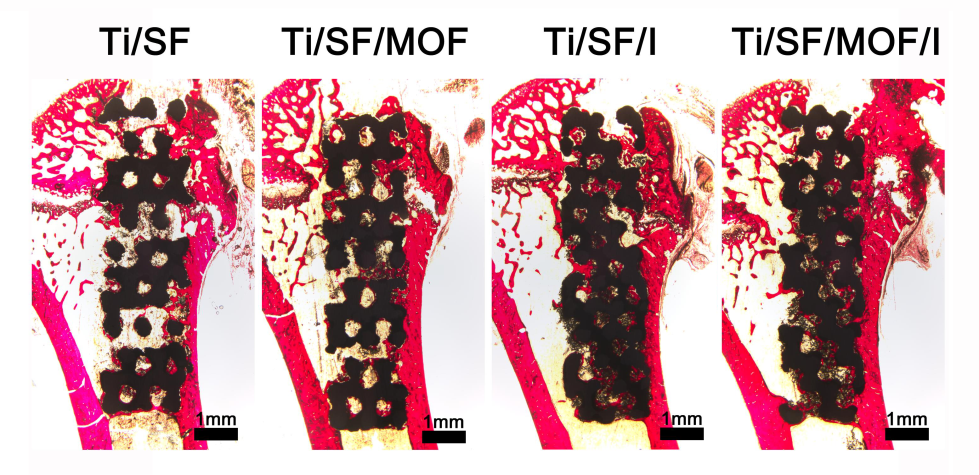


**Figure S6.** Van Gieson staining of undecalcified sections after samples implantation 8 weeks in normal female SD rats.
